# Exploring the utility of retinal optical coherence tomography as a biomarker for idiopathic intracranial hypertension: a systematic review

**DOI:** 10.1007/s00415-024-12481-3

**Published:** 2024-06-10

**Authors:** Mallika Prem Senthil, Saumya Anand, Ranjay Chakraborty, Jose Estevez Bordon, Paul A. Constable, Shannon Brown, Dalia Al-Dasooqi, Simu Simon

**Affiliations:** 1 College of Nursing and Health Sciences, Caring Futures Institute, Flinders University, Bedford Park, Adelaide, South Australia 5042 Australia; 2 Central Library, Flinders University, Bedford Park, Adelaide, South Australia Australia; 3 University of Adelaide, Adelaide, South Australia Australia

**Keywords:** Idiopathic intracranial hypertension, Optical coherence tomography, Optical coherence tomography angiography, Retinal nerve fiber layer, Optic nerve head, Peripapillary vessel density

## Abstract

This study aimed to examine the existing literature that investigated the effectiveness of optical coherence tomography (OCT) and optical coherence tomography angiography (OCT-A) as a biomarker for idiopathic intracranial hypertension (IIH). Our search was conducted on January 17th, 2024, and included the databases, Medline, Scopus, Embase, Cochrane, Latin American and Caribbean Health Sciences Literature (LILACS), International Standard Randomized Controlled Trial Number (ISRCTN) registry, and the International Clinical Trials Registry Platform (ICTRP). Our final review included 84 articles. In 74 studies, OCT was utilized as the primary ocular imaging method, while OCT-A was employed in two studies including eight studies that utilized both modalities. Overall, the results indicated that IIH patients exhibited significant increases in retinal nerve fiber layer (RNFL) thickness, total retinal and macular thickness, optic nerve head volume, and height, optic disc diameter and area, rim area, and thickness compared to controls. A significant correlation was observed between cerebrospinal fluid (CSF) pressure and OCT parameters including RNFL thickness, total retinal thickness, macular thickness, optic nerve head volume, and optic nerve head height. Interventions aimed at lowering CSF pressure were associated with a substantial improvement in these parameters. Nevertheless, studies comparing peripapillary vessel density using OCT-A between IIH patients and controls yielded conflicting results. Our systematic review supports OCT as a powerful tool to accurately monitor retinal axonal and optic nerve head changes in patients with IIH. Future research is required to determine the utility of OCT-A in IIH.

**Supplementary Information:**

The online version contains supplementary material available at 10.1007/s00415-024-12481-3.

## Introduction

Idiopathic intracranial hypertension (IIH) is a disorder of unknown etiology characterized by raised intracranial pressure (ICP) that predominantly affects obese women of childbearing age [1]. The prevalence of IIH in the general population is 1–3 per 100,000 people but among women of childbearing age, the prevalence rate is higher at 5.5 per 100,000 [2–5]. The incidence of IIH has increased due to the rapid increase of obesity and the estimated total cost of IIH in the USA alone has exceeded USD $444 million [6]. Although the exact cause of IIH is unknown, several theories have been postulated, including increased abdominal pressure, sleep apnea syndrome, reduction in cerebrospinal fluid (CSF) outflow or elevated venous sinus pressure [7–9]. The predominant symptom of IIH is headache, which can vary in intensity from mild to severe [10, 11] and chronic headache has been shown to significantly impact the quality of life of individuals with IIH [12, 13]. Other symptoms of IIH include tinnitus, visual obscuration, and diplopia [1, 10, 14, 15]. The accepted criteria for diagnosis of IIH includes the combination of raised ICP without hydrocephalus or mass lesions, normal CSF composition, and normal neuroimaging [13]. There are currently no evidence-based guidelines for the medical and surgical management of IIH due to a lack of information on the efficacy of treatments and possible side effects [16, 17].

Chronic elevated ICP can lead to cerebral ischemia, cerebral edema, herniation, irreversible brain damage and in severe cases, death [18]. Hence, the precise measurement and continuous monitoring of ICP are crucial in caring for patients with IIH [19]. ICP can be accurately assessed using lumbar and transcranial methods but are invasive and carry an increased risk of bleeding and infection [20, 21]. Several non-invasive methods such as magnetic resonance imaging, computerized axial tomography imaging, transcranial doppler ultrasonography, tympanic membrane displacement, and ocular ultrasound have also been used to monitor the ICP changes in patients with raised IIH. However, these methods have limitations such as low sensitivity and specificity, poor inter-rater reliability, and poor test predictability [22]. Therefore, more accurate and reliable biomarkers are needed to evaluate the disease state.

Optical coherence tomography (OCT) and optical coherence tomography angiography (OCT-A) are imaging modalities that provides qualitative and quantitative evaluation of the changes in the retinal nerve fibre layer (RNFL), optic nerve head, macula, and retinal and choroidal perfusion. While OCT can provide structural information about the retina and choroid, OCT-A provides information about the vasculature and blood flow of the retina and choroid (Table 1). OCT/OCT-A are routinely used in ophthalmic clinical settings to diagnose and monitor retinal conditions such as diabetic retinopathy, age-related macular degeneration, and retinal vascular occlusions [23–29]. OCT allows visualization of optic nerve head swelling, changes in the RNFL and retinal pigment epithelium/Bruch’s membrane (RPE/BM) layer associated with acute and chronic changes in ICP. In addition, OCT-A allows the evaluation of vessel density on the optic disc and peripapillary region in both newly diagnosed and chronic IIH cases, making these imaging modalities valuable tools for both diagnosing and monitoring IIH [30, 31]. OCT can be useful to differentiate true disc edema, including papilledema from pseudoedema due to optic disc drusen. Studies have shown that RNFL thickness particularly in the nasal and inferior quadrants were reduced in optic disc drusen compared to optic disc edema [32, 33]. The standard for evaluating the severity of papilledema is the Frisén scale which grades the optic disc swelling from 0 to 5. Three-dimensional OCT parameters such as optic nerve head volume, height, and shape could potentially offer greater sensitivity compared to the Frisén scale in evaluating treatment outcomes among IIH patients [34]. This is because even in IIH patients with normal RNFL thickness, the optic nerve head volume has been shown to be elevated [35]. The configuration of the RPE/BM layer in OCT scans can aid in distinguishing papilledema from disc edema caused by other factors like anterior ischemic optic neuropathy (AION). In papilledema, the RPE/BM layer exhibits a U-shape, angled toward the vitreous, whereas in AION and normal individuals, it assumes a V-shaped configuration, angled away from the vitreous [36, 37]. Increased ICP in IIH can cause biomechanical stress on the optic nerve head and retina resulting in retinal and choroidal folds, and OCT has been shown to be more sensitive than fundus photography is detecting these folds [38]. Quantitative assessments of vessel density surrounding the optic nerve head have shown a reduction in disorders such as optic neuritis, arteritic anterior ischemic optic neuropathy (AAION), and optic atrophy [39]. As such, the usefulness of OCT-A in diagnosing and monitoring IIH is still unclear. This systematic review examined the current body of literature regarding the utilization of OCT/OCT-A as a biomarker for IIH and reports the most suitable OCT/OCT-A parameters for the diagnosis and monitoring of IIH.

**Table 1:** Comparison of optical coherence tomography (OCT) and optical coherence tomography angiography (OCT-A) imaging techniques

	Optical coherence tomography (OCT)	Optical coherence tomography angiography (OCT-A)
Key components	It is a three-dimensional technique Optical coherence tomography uses infrared light directed toward the tissue under examination. The reflected light waves are then analyzed for time delay and differences in signal strength, which allows for assessment of tissue depth and imaging at the selected location The axial resolution of various OCTs is between 5 and 20 μm in the tissue	It is a three-dimensional technique Optical coherence tomography angiography uses low coherence interferometry with a focus on motion contrast of red blood cells for vascular imaging. The differences in the reflected OCT signal strength from consecutive OCT B-scans captured at the same location are used to generate an image of the blood flow The resolution of the instrument is 5–10 μm in the axial direction and ∼20 μm in the transverse direction
Strengths	Cross-sectional retinal imaging providing detailed imaging of the retinal, choroidal, and optic nerve head structure Rapid image generation, with processing completed in seconds Non-invasive Portable	Detailed imaging of the retinal, choroidal, and optic nerve head vasculature and perfusion Quantitative imaging on vascular density and perfusion Rapid image generation, with processing completed in seconds Enables imaging of the retinal vascular flow without the need for injection of a dye Non-invasive Portable
Limitations	Inability to image blood flow Motion artifacts during imaging can lower the quality of the image	It lacks the ability to show vascular leakage Small field of view Presence of artifacts can hinder accurate interpretation of the image
Image		

## Methods

This systematic review followed the reporting guidelines outlined in the Preferred Reporting Items for Systematic reviews and Meta-Analyses (PRISMA) and was registered with the International Prospective Register of Systematic Reviews (PROSPER; ID: CRD42024520282). An initial search was performed using Medline and CINAHL to identify relevant articles and keywords. An extensive search strategy was then developed in Medline based on the identified keywords and index terms. The keywords used for the search included: “idiopathic intracranial hypertension”, “pseudotumor cerebri”, “pseudotumor syndrome”, “Nonne’s syndrome”, “otitis hydrocephalus”, “benign intracranial hypertension”, “non-infective serous meningitis”. The retrieved articles from the Medline search were evaluated to confirm the inclusion of key publications. The search strategy, including the keywords and index terms, were adapted for other bibliographic databases such as PsycINFO (via Ovid SP), Latin American and Caribbean Health Sciences Literature (LILACS) and Scopus (Elsevier) (Online Resource 1). Each database search strategy was run on 17 January 2024. In addition, grey literature sources were searched including the International Standard Randomized Controlled Trial Number (ISRCTN) registry and the International Clinical Trials Registry Platform (ICTRP). There were no limits applied to language, but studies were excluded if they were solely animal studies, case report/case series, editorials, reviews, or conference abstracts. The primary outcome was to report the retinal and optic nerve head changes using OCT/OCT-A in IIH patients. Two authors (MPS and JE) independently evaluated the titles and abstracts and then full-text reports for relevance utilizing Covidence (IBM, Detroit USA). Any discrepancies in the screening were resolved by mutual consensus between the authors. Reasons for exclusion of the studies were reported in each step of the review process. Included studies were assessed for quality according to the National Institutes of Health Quality Assessment Tool for observational cohort, case–control, cross-sectional, before–after studies with no control groups and controlled intervention studies. The assessment of case–control and before–after studies included 12 items scored as “yes”, “no”, or “other” (cannot determine, not applicable, not reported). The assessment of cohort, observational, and controlled intervention studies included 14 items that were again scored as “yes”, “no” or “other” (cannot determine, not applicable, not reported).

## Results

**Fig. 1:**
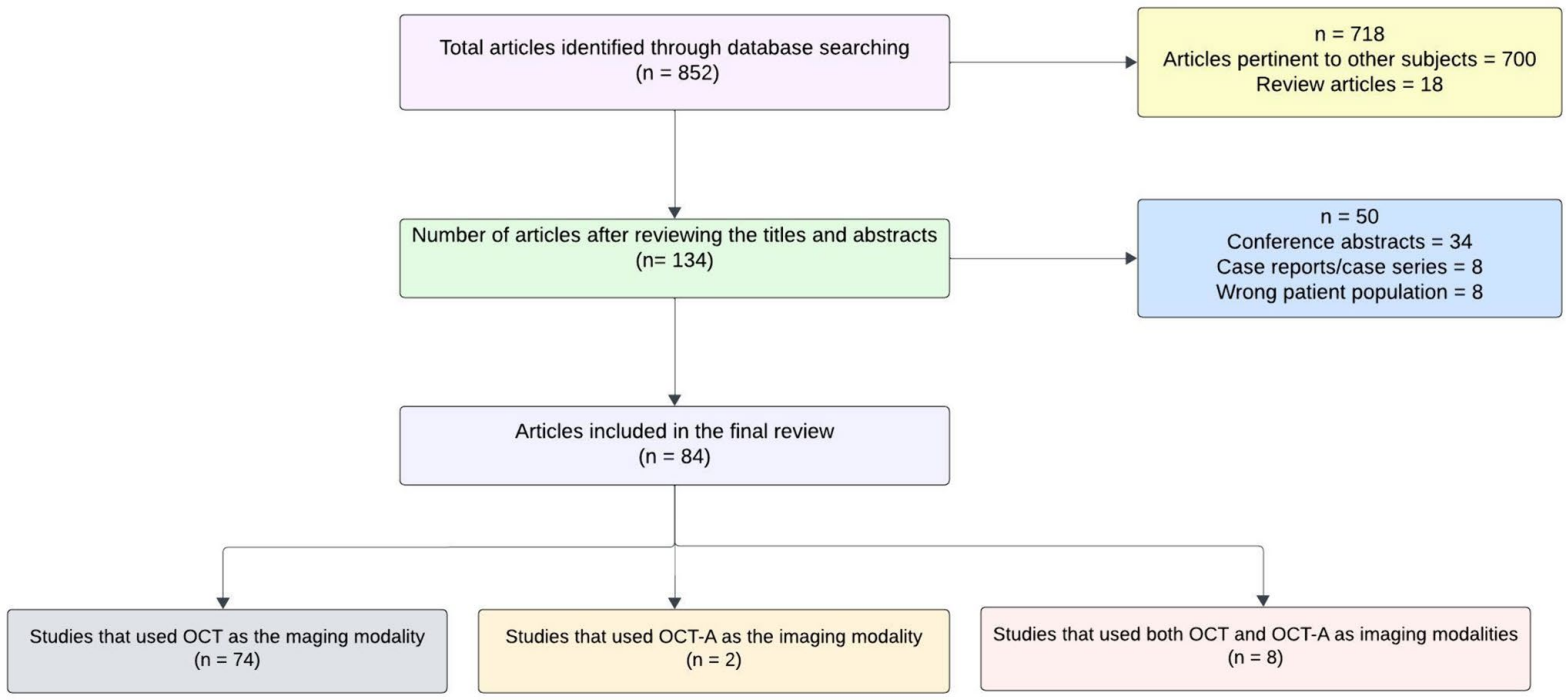
Flow diagram of the systematic literature review search

Our initial search yielded 852 articles. Upon reviewing titles and abstracts, 718 articles were excluded (Fig. 1). After examining the full text of the remaining articles, an additional 50 were excluded. The final review comprised 84 articles that utilized ocular imaging as a biomarker for IIH (Table 2). We categorized the included articles into two groups: (1) studies employing OCT as the imaging technique in IIH, and (2) studies employing OCT-A as the imaging technique in IIH. The assessment of the risk of bias of the studies included in this review is shown in Online Resource 2.

**Table 2:** Summary of the studies included in the systematic review

Author	Year	Country	Study design	Sample size	Imaging	Device	Results
Rebolleda et al. [40]	2009	Spain	Case–control	IIH = 22 Controls = 22	OCT	Stratus OCT	The mean retinal nerve fibre layer (RNFL) and RNFL thickness in all quadrants in the eyes with papilledema were significantly greater than that of controls
Jensen et al. [64]	2010	Denmark	Case–control	IIH = 20 Controls = 20	OCT	Stratus OCT 3000	Baseline average RNFL and retinal thickness were significantly higher in patients with IIH compared to control
Scott et al. [65]	2010	USA	Cross-sectional	Papilledema = 36	OCT	OCT3	There was a significant correlation between OCT RNFL thickness, OCT total retinal thickness and modified Frisén scale (MFS) grades from photographs
Sinclair et al. [93]	2010	UK	Cross-sectional	IIH = 25	OCT	Stratus OCT	Elevation of the optic disc, diameter of the optic nerve sheath, and thickness of peripapillary RNFL significantly improved after weight loss
Sibony et al. [36]	2011	USA	Case–control	Papilledema = 25 AION = 20 Controls = 30	OCT	Cirrus OCT	The retinal pigment epithelium-basement membrane (RPE/BM) layer in controls and anterior ischemic optic neuropathy has a V-shape pointing away from vitreous. However, papilledema has a U shape pointing toward vitreous. Weight loss and shunting moved the U shape to V shape in papilledema patients
Skau et al. [136]	2011	Denmark	Case–control	IIH = 20	OCT	Stratus OCT 3000	Peripapillary OCT is a promising objective examination modality for optic disc evaluation in IIH and may improve the identification of subtle disc swellings
Skau et al. [66]	2011	Denmark	Case–control	IIH = 17 Controls = 20	OCT	Stratus OCT 3000	At baseline, average RNFL thickness and retinal thickness were significantly higher in IIH compared to controls. Changes in visual field mean deviation and pattern deviation from baseline to the final visit correlated significantly with OCT changes. During follow-up, patients improved significantly in OCT parameters and visual field sensitivity
Waisbourd et al. [41]	2011	Israel	Cross-sectional	IIH = 48	OCT	OCT3 3000	Average RNFL thickness was statistically different between the groups: the normal optic disc/mild elevation group, moderate elevation group, and the full-blown papilledema group
Kaufhold et al. [35]	2012	Germany	Case–control	IIH = 19 Controls = 19	OCT	Spectralis OCT	The 3D parameters, optic nerve head volume (ONHV), and optic nerve head height (ONHH) were able to discriminate between controls, treated, and untreated patients. Both ONHV and ONHH measures were related to levels of intracranial pressure (ICP)
Yri et al. [67]	2012	Denmark	Case–control	IIH = 18 Controls = 20	OCT	Stratus OCT 3000	IIH patients with resolved papilledema at follow-up had a significant reduction in RNFL and retinal thickness values from 3-month follow-up visit to the final follow-up. At final follow-up, RNFL and retinal thickness were significantly thinner in IIH patients than healthy controls
Marzoli et al. [42]	2013	Italy	Cross-sectional	IIH = 38	OCT	RTVue-100 OCT	Mean average peripapillary retinal nerve fibre layer (ppRNFL) thickness was greater than normal values, while mean average macular ganglion cell complex thickness was lower than normal values
Skau et al. [43]	2013	Denmark	Case–control	IIH = 20 Controls = 20	OCT	Stratus OCT 3000	RNFL thickness was significantly increased in for all disc quadrants in newly diagnosed IIH
Auinger et al. [68]	2014	USA	Cross-sectional	IIH = 126 Controls	OCT	Cirrus OCT 4000	3D segmentation-based applications appear to be superior to commercially available 2D algorithms for calculating thickness of RNFL, total retinal thickness, and ganglion cell layer
Fard et al. [44]	2014	Iran	Case–control	Papilledema = 21 Pseudopapilledema = 19 Controls = 17	OCT	Spectralis OCT	RNFL thickness significantly increased in mild papilledema and pseudopapilledema compared to controls. Outer peripapillary total retinal volume is increased in only papilledema compared with pseudopapilledema and controls
Sibony et al. [19]	2014	USA	Pre–Post	IIH = 41 Controls = 30	OCT	Cirrus OCT	The ppRPE/BM changes with CSF pressure lowering interventions. Direct measurements of displacement at the basement membrane opening may serve as a more convenient office-based surrogate for shape analysis
Monteiro et al. [72]	2014	Brazil	Case–control	IIH = 29 Controls = 31	OCT	Cirrus OCT	The RNFL thickness and macular thickness significantly reduced in resolved papilledema compared to controls. Both measurements correlated with visual field sensitivity loss
OCT sub-study committee [34]	2015	USA	RCT	IIH = 89	OCT	Cirrus OCT 4000	RNFL thickness, total retinal thickness, and ONHV measurements improved with Diamox and weight loss
Chang et al. [89]	2015	USA	Case–control	IIH = 11 Controls = 11	OCT	Cirrus OCT	Mean OCT measurements of optic nerve protrusion length (NPL) were significantly larger than in controls. OCT and MRI measurements of NPL significantly correlated and significantly associated with Frisén papilledema grade
Chen et al. [76]	2015	USA	Cross-sectional	IIH = 31	OCT	Cirrus OCT 4000	A ganglion cell layer-inner plexiform layer (GCL-IPL) thickness less than or equal to 70 µm at initial presentation or progressive thinning greater than or equal to 10 µm with 2–3 weeks compared with baseline correlated with poor visual outcome
Afonso et al. [137]	2015	Brazil	Case–control	PTC = 24 Controls = 26	OCT	3D OCT-1000	Both pattern electroretinogram (PERG) N95 amplitude and OCT parameters were able to discriminate papilledema eyes from controls with a similar performance. There was a significant correlation between PERG amplitude values and OCT parameters
Goldhagen et al. [73]	2015	USA	Case–control	IIH = 43 Controls = 30	OCT	Spectralis OCT	Total macular thickness was significantly thinner within the fovea and inner macular ring in non-trophic papilledema vs controls
Labib et al. [77]	2015	Egypt	Case–control	IIH = 30	OCT	RTVue SD-OCT	Initial RNFL thickness was significantly higher whereas macular ganglion cell complex (GCC) was significantly lower than controls. Macular GCC and RNFL thickness correlated with visual field loss
Moss et al. [78]	2015	USA	Case–control	IIH = 10 Controls = 15	OCT	Spectralis OCT	Photopic negative response was decreased in IIH patients and correlated with chronic ganglion cell injury and clinical measure of acute optic nerve head pathology
Sibony et al. [94]	2015	USA	Cross-sectional	IIH = 165 eyes	OCT	Cirrus OCT 4000	Folds are common in IIH and are three basic patterns: peripapillary wrinkles, retinal folds, and choroidal folds. OCT, specifically the raster and en face imaging, appears to be more sensitive in detecting folds than color fundus photography
Starks et al. [45]	2016	USA	Cross-sectional	IIH = 13	OCT	Stratus OCT	The visual field pattern deviation and RNFL are improved after optic nerve sheath fenestration
Dinkin et al. [46]	2017	USA	Pre–Post	IIH = 13	OCT	Cirrus OCT	Stenting of venous sinus stenosis is safe and results in reduction of ICP in IIH patients. This is associated with improvement in papilledema, RNFL thickness, visual field parameters and symptoms
Gampa et al. [96]	2017	USA	Pre–Post	IIH = 20	OCT	Spectralis OCT	This study demonstrates a quantitative association between pp-BM shape and chronic ICP level. Changes in pp-BM shape is detectable within 1 h of lowering ICP. pp-BM shape may be a useful marker for chronic ICP level and acute ICP changes
Albrecht et al [86]	2017	Germany	Case–control	IIH = 21 Controls = 27	OCT	Spectralis OCT	In IIH, the ONHV increased and correlated with CSF pressure. The ONHV decreased after the initiation of treatment with Diamox
Kupersmith et al. [37]	2017	USA	Cross-sectional	IIH = 87	OCT	Cirrus OCT 4000	The various types of retinal folds associated with papilledema reflect biodynamic processes and show an acetazolamide treatment effect. Persistence of these folds, despite marked improvement in optic nerve head swelling, suggests permanent changes in the affected retinal tissues
Saenz et al. [47]	2017	USA	Cross-sectional	Papilledema = 23 Pseudopapilledema = 28	OCT	Cirrus OCT 4000	Compared to pseudopapilledema, papilledema eyes showed larger mean optic nerve sheath diameter, thicker RNFL
Wang et al. [30]	2017	USA	Cross-sectional	IIH = 125	OCT	Cirrus OCT 4000	Mean changes of the pRPE/BM shape measure were significant and in the positive direction (away from the vitreous) for the acetazolamide, but not for the placebo group
Aojula et al. [48]	2018	UK	Case–control	IIH = 46 Controls = 14	OCT	Spectralis OCT	Significantly greater thickness automated segmentation error (SegE) was present in RNFL thickness total area, assessed using ImageJ, in IIH patients compared to controls
Park et al. [79]	2018	USA	Case–control	IIH = 11 Controls = 11	OCT	Spectralis OCT	Mean full field photopic negative response (ffPhNR) amplitude was reduced significantly in the patients compared to controls. The pattern pERG amplitude correlated significantly with Humphrey visual field mean deviation and GCC volume. However, the fPhNR amplitude was not correlated significantly with Humphrey visual field mean deviation or GCC volume
Sheils et al. [87]	2018	USA	Cross-sectional	IIH = 126 Controls	OCT	Cirrus OCT	Correlations between disc area and optic nerve head volume were similar in the treatment groups at baseline but were weaker in the acetazolamide group compared with the placebo group at 6 and 12 months in study eyes
Banki et al. [97]	2019	USA	RCT	IIH = 165	OCT	Cirrus OCT 4000	The change in CSF pressure did not correlate with the change in IOP for either treatment group. There was no correlation between the CSF pressure and the optic nerve head (ONH) shape or CSF pressure-IOP pressure and ONH shape at baseline or at 6 months
Eren et al. [90]	2019	Turkey	Case–control	IIH = 54 Controls = 48	OCT	RTVue SD-OCT	The mean RNFL thickness was greater in the control group compared to IIH patients. The disc area, rim area was higher in the IIH group compared to controls, but the cup volume was lesser in the IIH group. There was a positive correlation between papilledema grade, rim area, RNFL thickness, CSF opening pressure, disc area, and rim area
Huang-Link et al. [49]	2019	Sweden	Case–control	IIH with papilledema = 8 IIH without papilledema = 9 Other neurological diseases = 19	OCT	Cirrus OCT 4000	IIH patients with papilledema had increased RNFL compared to IIH without papilledema and neurological diseases. RNFL reduced after CSF removal at 3 and 6 months in IIH patients with papilledema. However, no significant change in RNFL thickness after CSF removal was observed in IIH without papilledema or in patients with other neurological diseases
Onder et al. [50]	2019	Turkey	Cross-sectional	IIH = 18	OCT	Cirrus OCT 5000	There was a significant correlation between LP opening pressure and RNFL thickness. No association between RNFL measurements and MRI signs. Patients with IIH showed increased rim area and rim thickness but reduced optic cup volume
Pasaoglu et al. [99]	2019	Turkey	Case–control	IIH = 8 Controls = 10	OCT	Spectralis OCT	There was a significant difference in the anterior lamina cribrosa surface depth and the posterior lamina cribrosa surface depth between IIH patients and controls. No significant difference in lamina cribrosa thickness between IIH and controls
Merticariu et al. [51]	2019	Romania	Case–control	IIH = 11 Controls = 13	OCT	DRI Oct Triton	There was a significant difference in the average RFNL thickness between IIH and controls. There was also a correlation between papilledema severity and RNFL thickness
Wall et al. [52]	2019	USA	Case–control	IIH = 39 Controls = 98	OCT	Cirrus 5000 OCT	Although the presence of papilledema limited correlation, 55% of the temporal wedge defects had OCT RNFL deficits in the corresponding superonasal location
Vijay et al. [88]	2020	UK	Cross-sectional	IIH = 104	OCT	Spectralis OCT	ONHV on OCT correlated with ICP. OCT can be used as a surrogate to inform ICP changes
Chen et al. [80]	2020	China	Cross-sectional	Primary PTCS = 9 Secondary PTCS = 46	OCT	Cirrus OCT	Secondary pseudotumor cerebri syndrome (PTCS) is more common than primary PTCS. OCT showed that eyes with intact macular GCL-IPL at baseline had good outcomes after treatment
Dreesbach et al. [103]	2020	Germany	Case–control	IIH = 21 Control eyes = 25	OCT	Spectralis OCT	ONHV was significantly increased in IIH compared to controls. The Frisén scale grading correlated higher with the ONHV than with RNFL thickness
Bahnasy et al. [53]	2020	Egypt	Cross-sectional	IIH with medical treatment = 59 IIH with LPS = 9	OCT	Spectralis OCT	Patients needed lumboperitoneal shunt showed statistically significant increase in baseline papilledema grade, mean deviation of visual field examination, optic nerve sheath diameter, average OCT–RNFL thickness, and P100 pattern reversal visual evoked potential latency. On the other hand, both studied groups showed statistically nonsignificant differences regarding the patients’ ages and opening CSF pressure
Tatar et al. [100]	2020	Turkey	Case–control	IIH = 15 Controls = 17	OCT	EDI-OCT	Prelaminar tissue thickness and Bruch’s membrane opening were significantly greater in IIH than controls. Anterior lamina cribrosa surface depth was significantly less in IIH than controls. No difference regarding lamina cribrosa thickness between IIH and controls
Ozdemir et al. [101]	2020	Turkey	Case–control	IIH = 22 Controls = 22	OCT	3D OCT-2000	IIH patients had increased RNFL thickness and choroidal thickness compared to controls. Subfoveal choroidal thickness was significantly correlated with ICP
Flowers et al. [54]	2021	USA	Cross-sectional	Papilledema = 22 Pseudopapilledema = 36	OCT	Cirrus OCT	The papilledema group had a higher mean RNFL thickness than the pseudopapilledema group
Nogueira et al. [81]	2021	Brazil	Case–control	IIH = 22 Controls = 11	OCT	RTVue-100	There was a significant association between macular GCC thickness and optic disc pallor and between edema and visual acuity. No significant difference was found in RNFL thickness between patients and controls. Macular GCC was thinner in patients with IIH compared to controls
Bingol Kiziltuncet et al. [69]	2021	Turkey	Cross-sectional	IIH = 28	OCT	RTVue XR OCT	Bruch’s membrane opening and maximal RNFL thickness were significantly higher in patients with increased CSF pressure. There exist correlations between CSF pressure and Bruch’s membrane opening, maximal RNFL thickness and maximal retinal thickness. Bruch’s membrane opening and maximal RNFL thickness can give an idea about increased CSF pressure values in IIH patients
Carey et al. [55]	2021	USA	Cross-sectional	IIH = 2 (20 scans)	OCT	Cirrus OCT	Multiple objective parameters of en face OCT of optic disc edema have an excellent correlation with ppRNFL thickness
Kabatas et al. [74]	2021	Turkey	Case–control	IIH = 56 Controls = 50	OCT	RTVue-XR 100 OCT	There was a significant difference in the mean RFNL thickness between IIH and controls. There was a significant positive correlation between the peripapillary RNFL thickness, the GCC thickness, and the CSF opening pressure. A similar result was also found between the Frisén grade and peripapillary RNFL
Kohil et al. [56]	2021	USA	Cross-sectional	Papilledema = 25 Pseudopapilledema = 24	OCT	Cirrus 5000 OCT	Ocular ultrasonography was 68% sensitive for papilledema and 54% specific for pseudopapilledema. Positive OUS correlated with elevated opening pressure on lumbar puncture and with signs of increased ICP on MRI
Panyala et al. [98]	2021	India	Case–control	Papilledema = 30 Papillitis = 30 Controls = 80	OCT	Cirrus OCT	97% of the eyes with papilledema had positive ppRPE/BM angle and 97% of the eyes with papillitis had negative ppRPE/BM angle. At 1 month, both RNFL thickness and ppRPE/BM reduced significantly in eyes with papilledema. RNFL normalized in 3 months and RPE/BM normalized in 6 months in patients with papilledema
Reggie et al. [95]	2021	USA	Cross-sectional	Papilledema = 32 Pseudopapilledema = 46	OCT	Cirrus 5000 OCT	Choroidal and/or retinal folds on OCT are commonly observed in patients with mild papilledema and are uncommon in those with pseudopapilledema
Touzé et al. [84]	2021	Canada	Cross-sectional	IIH = 16	OCT	Cirrus OCT	Mean RNFL was significantly decreased 1 month after stenting and at last visit. Ganglion cell thickness moderately decreased after stenting
Wibroe et al. [82]	2021	Denmark	Cross-sectional	IIH = 32	OCT	Spectralis OCT	The high prevalence of hyperreflective lines and peripapillary hyperreflective ovoid mass-like structures in IIH patients suggest these structures be a result of crowding in the optic nerve head caused by papilledema
Banerjee et al. [138]	2022	India	Case–control	IIH = 27	OCT	Cirrus OCT 4000	At baseline, average RNFL had a moderate negative correlation with mean deviation and a positive correlation with log MAR visual acuity. Baseline GCL and log MAR visual acuity had a negative correlation. Optic disc height (ODH) had a negative correlation with visual field mean deviation. At 6 months, ODH and GCL-IPL complex had a statistically significant correlation with functional parameters
Inam et al. [57]	2022	USA	Cross-sectional	IIH = 53	OCT	SD-OCT	The mean changes in OCT RNFL and mean deviation in Humphrey visual field at 6 weeks were not different between low, medium, and high venous sinus pressure gradient
Jacobsen et al. [7]	2022	Norway	Case–control	IIH = 20 Controls = 12	OCT	Nidek’s SD-OCT 3000	The peripapillary Bruch’s membrane angle (pBA) and the ONHH differed between the IIH and reference groups and correlated with both mean ICP wave amplitude and mean ICP
Thaller et al. [58]	2022	UK	Cross-sectional	IIH = 123	OCT	SD-OCT	Increased weight gain during lockdown was associated with significant increase in papilledema (RNFL). RNFL thickness was reduced in patients with weight loss
Rehman et al. [91]	2022	India	Cross-sectional	IIH = 30	OCT	Cirrus OCT	A statistically significant change was observed in all OCT parameters during the 6-month follow-up (RNFL thickness, disc area, rim area, ONHV, mean retinal thickness, choroidal thickness)
Rehman et al. [75]	2022	India	Cross-sectional	IIH = 10	OCT	Cirrus OCT	Statistically significant difference in RNFL thickness and macular thickness in nasal quadrant was seen between the eyes with recurrence and without recurrence
Sood et al. [59]	2022	India	Cross-sectional	Optic disc edema = 64 (IIH = 10)	OCT	Cirrus OCT	The clinical severity of optic disc edema correlated positively with RNFL thickness, and most of the categories of optic disc edema followed the normative pattern of RNFL thickness (inferior > superior > nasal > temporal) despite thickening
Vosoughi et al. [60]	2022	Canada	Cross-sectional	IIH = 186	OCT	Cirrus 5000 OCT	Patients seeking care due to symptoms of IIH also had higher RNFL thickness, worse visual function, a higher, LP opening pressure, and worse final visual outcome
Attia et al. [70]	2023	France	Cross-sectional	IIH = 49 Intracranial tumors = 33 Cerebral venous thrombosis = 15	OCT	Spectralis OCT	Mean RNFL thickness and retinal thickness was significantly different between the optic atrophy and no atrophy group
Kaya et al. [92]	2023	Turkey	Case–control	IIH = 43 Controls = 20	OCT	RTVue OCT	In the resolved-papilledema subgroup, peripapillary choroidal thickness in all quadrants was significantly lower than in the control group. In the acute-papilledema subgroup, peripapillary choroidal thickness in the temporal, inferior, and superior quadrants was significantly less than in the control eyes. The disc diameters in the vertical and horizontal planes was also significantly larger in the acute-papilledema eyes than in the control eyes and in the resolved papilledema eyes
Kaya Tutar et al. [61]	2023	Turkey	Cross-sectional	IIH = 21	OCT	Spectralis OCT	A statistically significant positive and moderate correlation was found between CSF pressure values and average RNFL thickness
Thaller et al. [139]	2023	UK	Cross-sectional	IIH = 490	OCT	Spectralis OCT	Those with the highest OCT RNFL had the worst visual outcomes
Thaller et al. [85]	2023	UK	Cross-sectional	IIH = 343	OCT	Spectralis OCT	OCT parameters did not differ significantly between symptomatic and asymptomatic disease
Xie et al. [62]	2023	Canada	Cross-sectional	IIH = 165	OCT	Cirrus OCT	Papilledema recurrence can be detected in atrophic optic discs using OCT
Bassi et al. [83]	2024	India	Cross-sectional	IIH = 20	OCT	SD-OCT	The RNFL thickness in all four quadrants had a weak positive correlation, and the GCL-IPL layer had a weak negative correlation with the ICP
Srija et al. [63]	2024	India	Case–control	Optic disc edema = 81 Controls = 74	OCT	Topcon 3D-OCT	Peripapillary RNFL thickness was significantly increased in the optic disc oedema group compared to controls
Wang et al. [71]	2024	USA	Case–control	IIH = 125 Controls = 96	OCT	Cirrus OCT	The montage colormaps of RNFL and total retinal thickness produced by the biVAE model provided an organized visualization of the variety of morphological patterns of optic disc oedema (including differing patterns at similar thickness levels)
Yalcinkaya Cakir et al. [108]	2023	Turkey	Case–control	IIH = 30 ODD = 33 Controls = 70	OCT & OCT-A	Topcon	Peripapillary vascular density is reduced in patients with IIH and optic disc drusen (ODD) compared to healthy controls. There is a significant difference in the vascular density in deep capillary plexus and choriocapillaris between IIH and ODD
Fard et al. [110]	2019	Iran	Case–control	Papilledema = 21 Pseudopapilledema = 15 Controls = 44	OCT & OCT-A	AngioVue	Average RNFL was greater in patients with papilledema compared to pseudo and controls. No difference in GCC thickness between the three groups. Peripapillary vasculature values were significantly lower in papilledema and pseudo compared to controls
Rodriguez Torres et al. [140]	2021	USA	Cross-sectional	IIH = 23	OCT & OCT-A	AngioVue	Skeletonized vessel density peripapillary capillary plexus was significantly associated with papilledema grades, RNFL thickness, GCL thickness. Increased grading was associated with decrease of vessel density
El-Haddad et al. [141]	2023	Egypt	Cross-sectional	IIH = 21	OCT-A	AngioVue	Optic disc vessel density decreased after shunt surgery in patients with IIH. There were positive correlations between the CSF opening pressures and the preoperative optic disc vessel density of the whole image and inside disc. In addition, there was a positive correlation between the opening CSF pressures and the reduction in whole image vessel density after surgery
Wang et l [111]	2023	China	Case–control	IIH = 61 Controls = 65	OCT & OCT-A	VG200S, Svision	Patients with IIH showed thicker ppRNFL and GCL-IPL thickness with larger ONH rim area when compared to controls. Microvascular densities were increased in nerve fibre layer plexus while densities were reduced in superficial vascular plexus, intermediate capillary plexus, and deep capillary plexus compared to controls
Pahuja et al. [102]	2023	India	Case–control	IIH = 41 Controls = 10	OCT & OCT-A	Angioplex	RNFL thickness showed significant thinning in the early, chronic, and atrophic papilledema. GC-IPL was significantly reduced in all groups compared to controls
Kwapong et al. [113]	2023	China	Case–control	IIH = 46 Controls = 42	OCT & OCT-A	VG200S, Svision	Compared with the control group, IIH patients showed reduced microvascular densities and thinner retinal thicknesses. ICP correlated with the microvascular densities and GCL-IPL thickness in IH patients for superficial vascular complex and deep vascular complex
Chonsui et al. [112]	2022	France	Cross-sectional	Papilledema = 20 Controls = 33	OCT & OCT-A	PLEX Elite	There was a decreased peripapillary capillary density without changes in capillary flux intensity (CFI) in eyes with papilledema. There were a positive association between the CFI and the RNFL and a negative association between the capillary perfusion density (CPD) and the RNFL
Kaya et al. [109]	2021	Turkey	Case–control	IIH = 31 Controls = 52	OCT-A	AngioVue	The vessel density in the inferior nasal region in IIH patients significantly exceeded the vessel density of the controls. RFNL and GCC thickness were comparable between IIH and controls
Tüntaş Bilen et al. [107]	2019	Turkey	Case–control	IIH = 19 Controls = 21	OCT & OCT-A	AngioVue	There was a decrease in peripapillary density in patients with IIH compared to health controls

*RCT* randomized controlled trail; *IIH* idiopathic intracranial hypertension; *AION* anterior ischemic optic neuropathy; *PTC* pseudotumor cerebri; *PTCS* pseudotumor cerebri syndrome; *LPS* lumboperitoneal shunt; *ODD* optic disc drusen; *OCT* optical coherence tomography; *OCT-A* optical coherence tomography angiography; *CSF* cerebrospinal fluid; *ICP* intracranial pressure

### Studies investigating OCT as the imaging modality in IIH

**Fig. 2:**
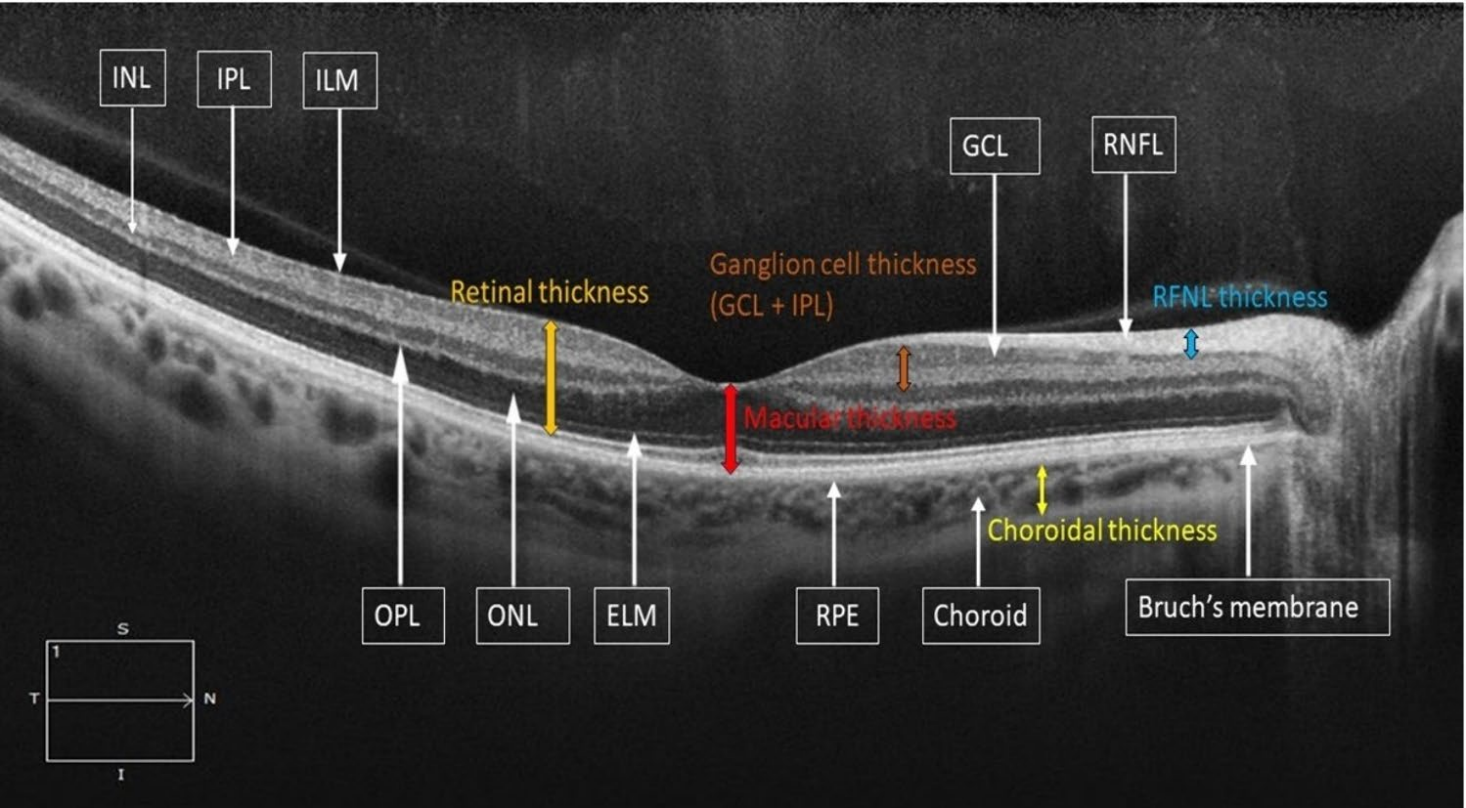
Optical coherence tomography (OCT) image of the retina showing the different layers. *ILM* inner limiting membrane; *RNFL* retinal nerve fiber layer; *GCL* ganglion cell layer; *INL* inner nuclear layer; *IPL* inner plexiform layer; *ONL* outer nuclear layer; *OPL* outer plexiform layer; *ELM* external limiting membrane; *RPE* retinal pigment epithelium. Macular thickness = distance between ILM and RPE; retinal thickness = distance between ILM and photoreceptor layer; choroidal thickness = distance between the outer border of RPE and choroidoscleral surface

**Fig. 3:**
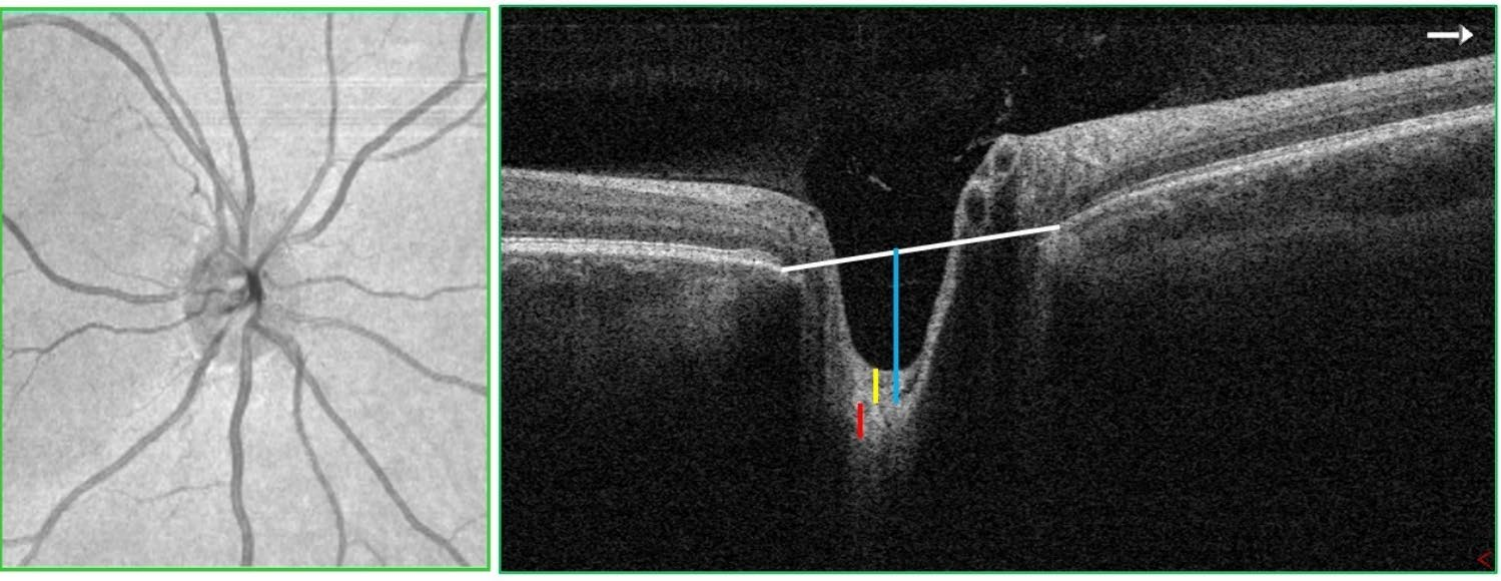
Optical coherence tomography (OCT) imaging of the optic disc. The white line is Bruch’s membrane opening, the blue line is lamina cribrosa surface depth, the yellow line is pre-laminar tissue thickness, and the red line is lamina cribrosa thickness

**Fig. 4:**
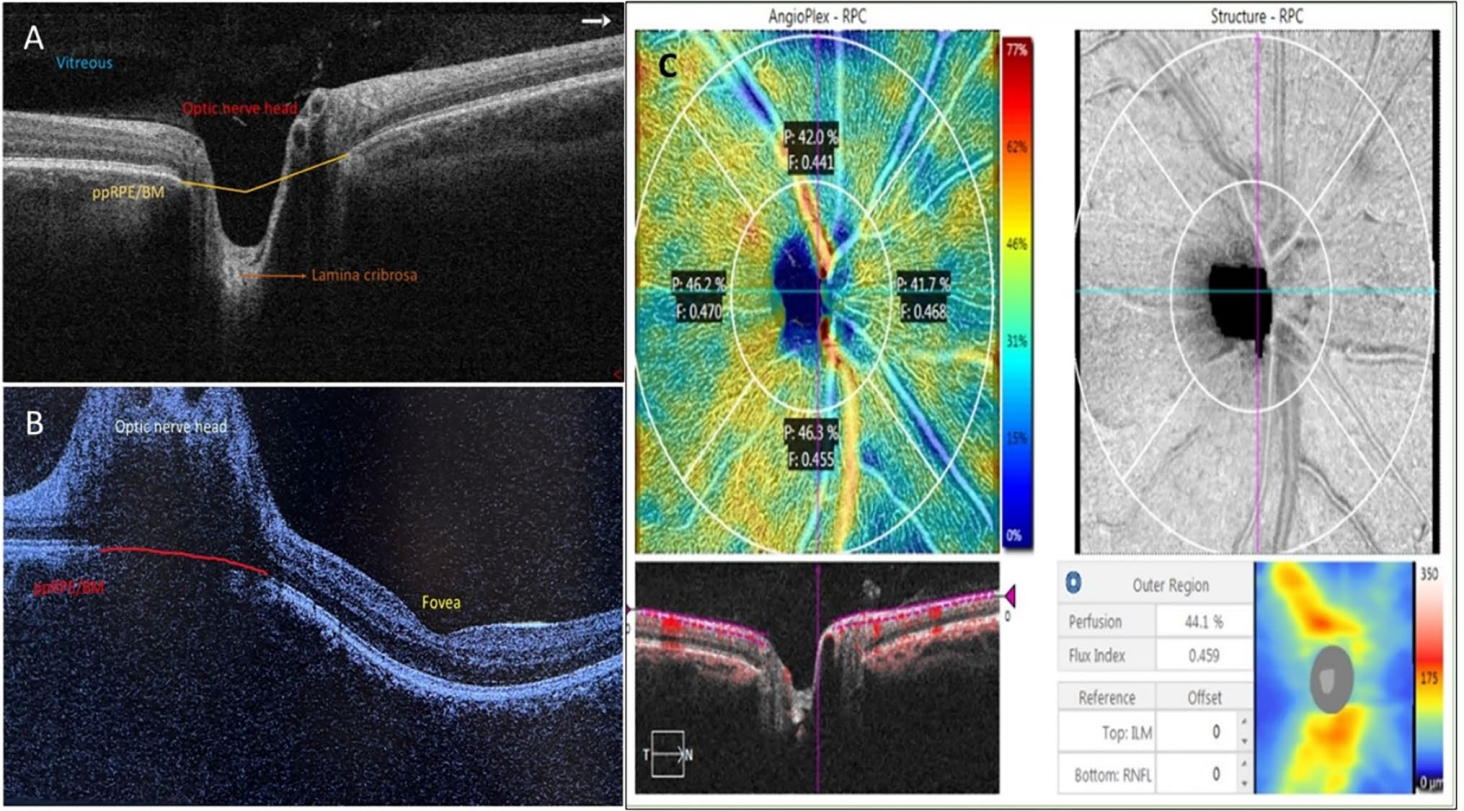
**A** Optical coherence tomography (OCT) image of the optic nerve head. In healthy individuals, the peripapillary retinal pigment epithelium-basement membrane (ppRPE/BM) is V-shaped and angled away from the vitreous. **B** Optical coherence tomography (OCT) image of the optic nerve head showing a U-shape configuration of the peripapillary retinal pigment epithelium-basement layer (ppRPE/BM) in a patient with papilledema. **C** Optical coherence tomography angiography (OCT-A) image of the optic nerve head

**Fig. 5:**
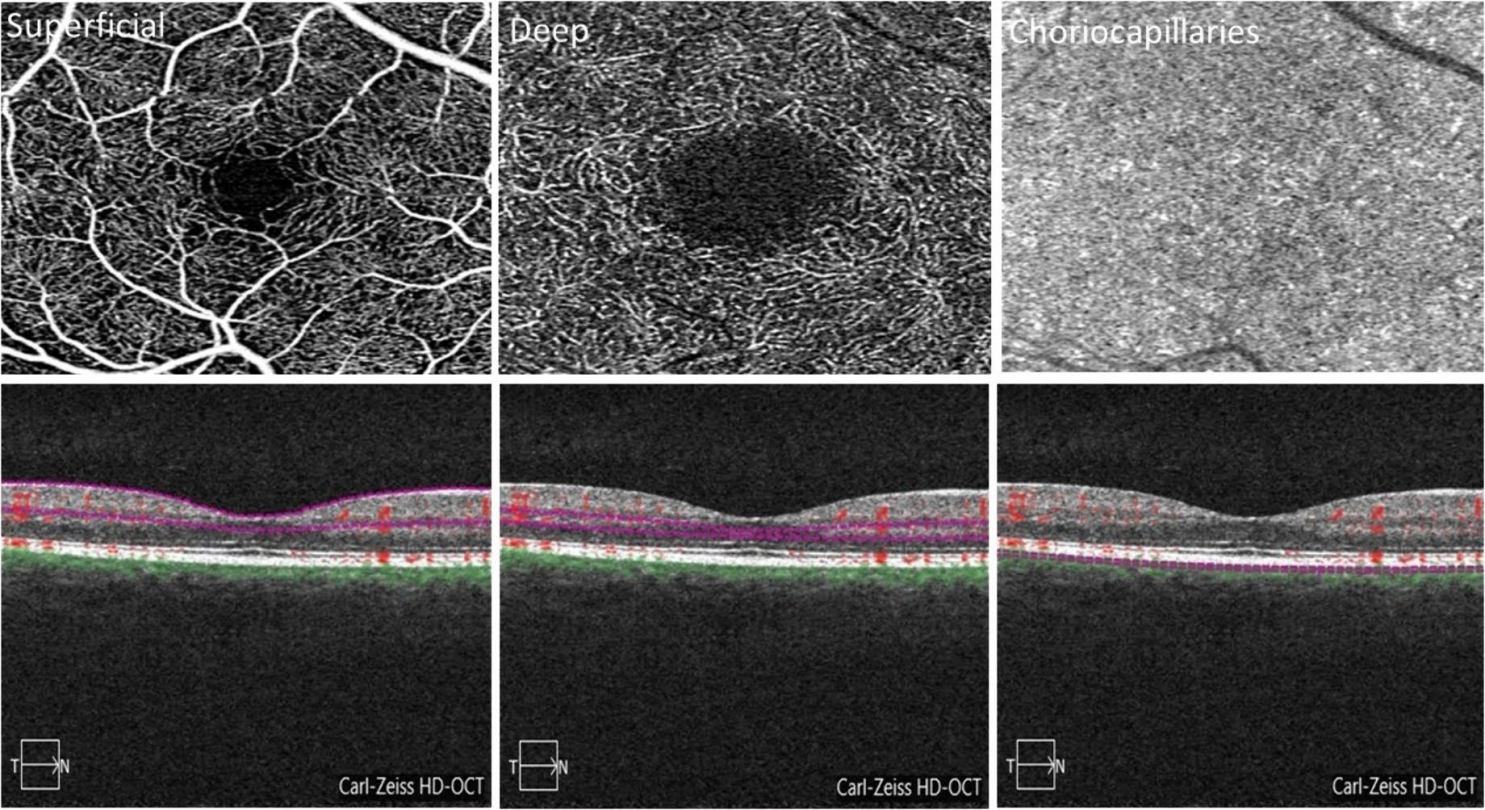
Optical coherence tomography angiography (OCT-A) image displays the segmentation of three capillary plexus, including superficial, deep, and choriocapillary plexus, both in en face (top row) and cross-sectional (bottom row). The segmentation boundaries for each layer are indicated by a pink line on the cross-sectional OCT-A image

There were 82 studies that used OCT as an imaging modality in IIH. In these studies, OCT imaging was used to evaluate treatment effectiveness in patients with IIH, to compare the retinal and optic nerve head changes between IIH patients and healthy controls or to ascertain the relationship between OCT measurements and clinical parameters. The following OCT parameters were compared among the studies that reported them: peripapillary RNFL thickness [40–63], total retinal thickness [34, 64–71], macular thickness [72–75], macular ganglion cell complex (GCC) thickness [76–83], ganglion cell layer thickness [70, 84, 85], optic nerve head shape [30], optic nerve head volume [34, 35, 86–88], optic nerve head height [7, 35], optic nerve protrusion length [89], optic disc area [90, 91], optic disc diameter [92, 93], rim area [90, 91], rim thickness [50], optic cup volume [90], retinal folds [37, 94, 95], shape of peripapillary retinal pigment epithelium-basement membrane (ppRPE/BM) layer [7, 19, 30, 36, 96–98], anterior laminar cribrosa surface depth [99], posterior lamina cribrosa surface depth [99, 100], lamina cribrosa thickness [99, 100], Bruch’s membrane opening [69, 100], and pre-laminar tissue thickness (Figs. 2 and 3) [100]. The RNFL thickness was the OCT parameter most used in these studies [40, 43, 64, 66]. Most studies that compared the RNFL thickness between IIH patients and age-matched controls demonstrated that IIH patients had a significantly greater RNFL thickness compared to the controls [40, 43, 44, 51, 63, 64, 66, 101]. However, one study contradicted this trend, suggesting that control subjects had higher RNFL thickness compared to those with IIH, while another study found no significant difference in RNFL thickness between IIH patients and controls [81, 90]. In addition, it was observed that that IIH patients had initially thicker RNFL measurements, which gradually decreased over subsequent follow-up periods at 1, 3, 6, and 12 months [34, 40, 66, 67]. Among the IIH patients, those with severe papilledema were shown to have thicker RNFL than in patients with normal optic discs/minimally or moderately raised discs [41]. Patients with recurrent IIH and those without recurrence of IIH were found to have significantly different RNFL thicknesses, with the recurrence group reported to have thicker neural tissue [91]. Similarly, RNFL was greater in papilledema than in pseudopapilledema patients [54]. However, the RNFL thickness did not differ between the symptomatic and asymptomatic groups [85]. Compared to healthy controls, patients with chronic and atrophic papilledema had significantly thinner RNFL thickness [102]. In OCT imaging, ppRPE/BM is seen as a well-defined layer above the choroid in the outer retina. It is V-shaped and angled away from the vitreous in normal individuals (Fig. 4). However, in IIH patients with raised ICP, it is U-shaped and angled toward the vitreous (Fig. 5) [36, 98]. Interventions aimed at lowering the CSF pressure such as lumbar puncture and CSF shunt have demonstrated an ability to transform the ppRPE/BM layer from a U-shaped configuration to the more typical V-shaped configuration [19, 30, 36].

Few studies used a custom segmentation algorithm to develop 3D parameters such as optic nerve head volume and optic nerve head height to compare the optic nerve head changes between IIH patients and controls. They showed that the optic nerve head volume and optic nerve head height were increased in IIH patients than in controls [35, 103]. In addition, optic nerve head volume was used to differentiate between controls, treated, and untreated patients with IIH [35]. Optic disc area, diameter, rim area, thickness, and Bruch’s membrane opening were reported to be thicker in IIH patients compared to controls [69, 90]. In contrast, individuals with IIH exhibited thinner macular GCC, diminished thickness of the peripapillary choroid, reduced depth of both the anterior and posterior lamina cribrosa surfaces depths, and a decrease in optic cup volume [42, 77, 81, 90, 99]. OCT imaging was also shown to be sensitive in detecting folds such as peripapillary wrinkles, retinal and choroidal folds in patients with IIH [95].

Studies that investigated the utility of OCT in evaluating treatment outcomes in IIH patients demonstrated a significant improvement in the OCT parameters (RNFL thickness, total retinal thickness, choroidal thickness, optic nerve head volume, rim, and disc area) after interventions such as weight loss, oral acetazolamide, and optic nerve sheath fenestration [45, 86, 91]. Studies that examined the relationship between OCT measurements and clinical parameters showed a significant positive correlation between CSF pressure and various OCT parameters, including RNFL thickness, retinal thickness, macular GCC, optic nerve head volume, optic nerve head height, ppRPE/BM layer and Bruch’s membrane opening [35, 50, 51, 59, 61, 69, 86]. RNFL thickness showed significant positive correlation with visual acuity, visual field loss, papilledema severity, and the Modified Frisén Scale (MFS grades) from fundus photographs [40, 65, 77]. In addition, macular GCC thickness was found to be significantly associated with optic disc pallor [81]. However, no association was identified between CSF pressure and the shape of the optic nerve head [97].

### Studies investigating OCT-A as the imaging modality in IIH

Ten studies explored the applicability of OCT-A as a non-invasive imaging biomarker for IIH. In these studies, OCT-A examinations were primarily done in acute settings of papilledema except two studies in chronic papilledema settings. Most of these studies employed OCT-A to assess the peripapillary vascular density differences between individuals with IIH and control groups. The peripapillary area is a ring-shaped zone extending from the optic disc boundary (Fig. 4) [104]. Vessel density is defined as the percentage of area occupied by both large vessels and microvasculature in a specific area and is calculated over the entire scan area, as well as in the defined sectors within the scan [105]. Capillary flux intensity is defined as the total weighted area of perfused microvascular per unit area and capillary perfusion density is defined as the total area of perfused microvasculature per unit area. The retinal vascular network is organized into four distinct plexuses: the superficial capillary plexus (SCP), intermediate capillary plexus (IP), deep capillary plexus (DCP), and radial peripapillary capillary plexus (RPC) [106]. The central retinal artery supplies blood to the SCP which then anastomoses and creates the IP and the DCP. The SCPs are located with the RNFL, ganglion cell layer, and the inner plexiform layer and the DCPs are located within the outer plexiform layer below the IP. The RCP, however, runs parallel with the nerve fiber layer axons (Fig. 5).

Several studies have investigated the peripapillary vessel density in patients with IIH in comparison to controls. Tuntas et al. found a significant reduction in peripapillary vessel density among IIH patients compared to controls in their study using the AngioVue OCT-A device, which exclusively reported peripapillary vessel density (both global and sectoral). [107] Similarly, Cakir et al., utilizing Topcon imaging, observed a notable decrease in peripapillary vessel density across different retinal capillary plexus (SCP, DCP, and choriocapillaries) in IIH patients compared to controls [108]. However, Kaya et al., also using AngioVue reported a significant elevation in peripapillary vessel density in IIH patients compared to controls, offering a contradictory perspective [109]. In contrast, Fard et al., employing AngioVue found no significant difference in peripapillary capillary density between papilledema patients and controls [110]. Moreover, microvascular densities showed an increase in the nerve fiber layer plexus (NFLP) but a reduction in the SCP and DCP in IIH patients compared to controls using the Svision imaging OCT-A device [111]. Chonsui et al.in their study using PLEX Elite device showed a decreased peripapillary capillary density without changes in capillary flux intensity in eyes with papilledema [112].

Wang et al.in their study using AngioVue device showed that NFLP positively correlated with Frisén scores of patients with IIH [111]. However, SVP, IP, and DCP inversely correlated with Frisén scores of patients with IIH. Similarly, Pahuja et al. showed a negative correlation between superficial peripapillary retinal vessel perfusion and grades of papilledema using the Angioplex device (reported superficial capillary retinal vessel perfusion, deep retinal vessel perfusion and peripapillary choriocapillary perfusion) [102]. Kwapong et al. showed microvascular densities (superficial vascular complex and deep vascular complex) positively correlated with ICP using the Svision OCT-A device [113]. Peripapillary capillary vessel density in DCP was significantly reduced in optic disc edema compared to the control group, a condition that can mimic IIH. [108]

## Discussion

OCT and OCT-A are non-invasive imaging methods widely used in ophthalmology to provide high-resolution cross-sectional images of the retina [114, 115]. OCT and OCT-A measurements have also shown to be a reliable indicator of neuronal death in various neurological disorders such as Parkinson’s disease, Alzheimer’s disease, multiple sclerosis, neuromyelitis optica, and spinocerebellar ataxia [116–119]. This systematic review examined existing literature to assess the effectiveness of OCT/OCT-A as a diagnostic and monitoring modality for IIH. The predominant imaging technique in the reviewed studies was OCT, with only ten studies using OCT-A. Among studies using OCT as the imaging modality for IIH patients, the most frequently assessed parameter was RNFL thickness. Conversely, studies employing OCT-A as the imaging modality for IIH patients predominantly focused on peripapillary vessel density. In summary, studies utilizing OCT revealed increased thickness in RNFL, retina, as well as increased measurements in optic nerve head volume, optic nerve head height, optic disc diameter, rim area, and rim thickness. However, studies that used OCT-A as the imaging modality showed conflicting results regarding the peripapillary vessel density.

**Fig. 6:**
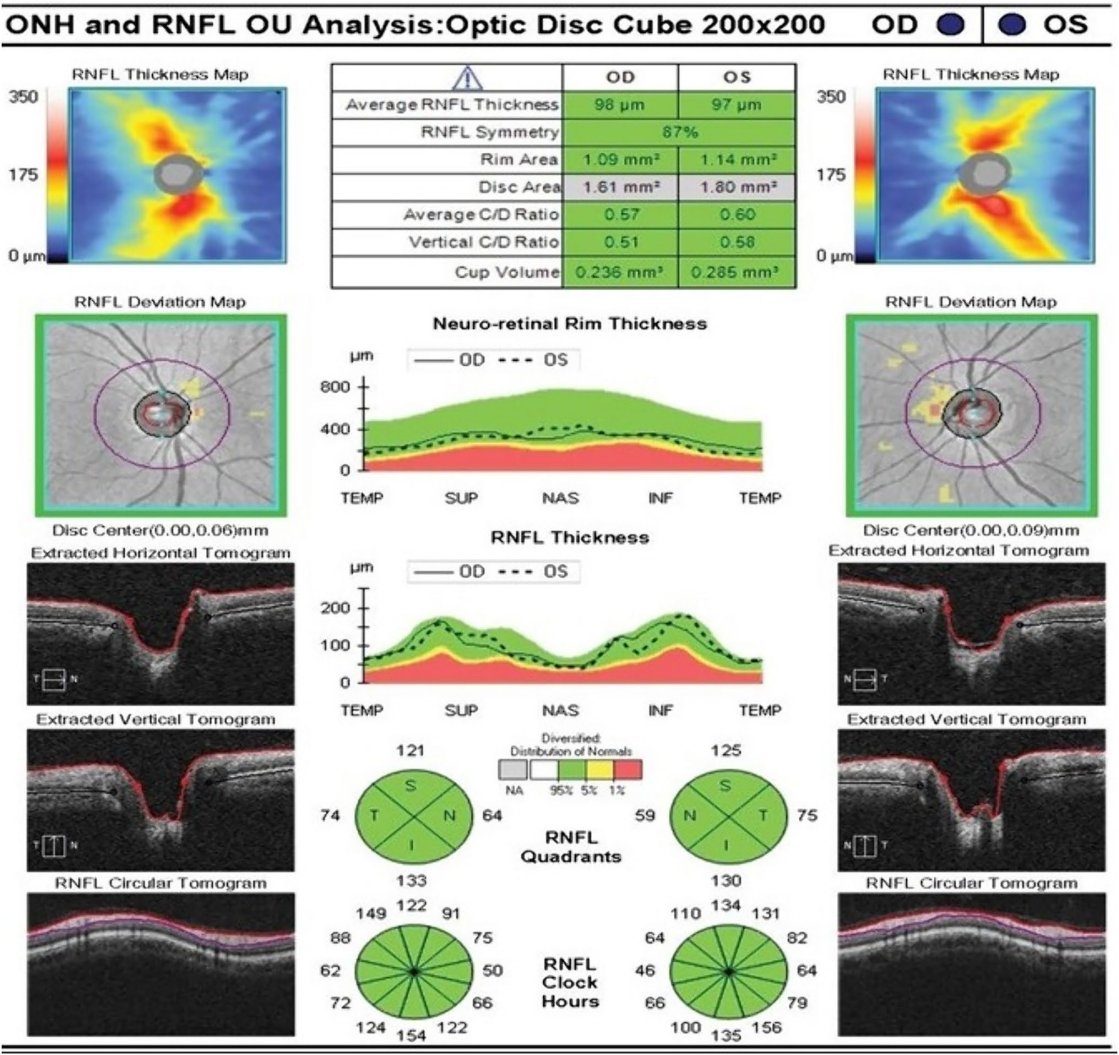
Optical coherence tomography (OCT) test results showing optic nerve head (ONH) and retinal nerve fibre layer (RFNL) thickness of right (OD) and left eye (OS)

The RNFL comprises axons originating from retinal ganglion cells that converge from the retina and macular region to form the optic nerve. The RNFL is visualized in OCT images as the inner most retinal layer beneath the internal limiting membrane (Fig. 2). The peripapillary RNFL is measured along a 3 mm diameter circle centered on the optic nerve head and the mean thickness of the upper retinal layer is then presented as the average RNFL thickness (Fig. 6). The increased RNFL thickness reported in individuals with IIH is due to the disruption of the axonal transport and intraneural optic nerve sheath ischemia caused by the elevated CSF pressure in the subarachnoid space surrounding the optic nerve. Conversely, the reported decrease in macular GCC thickness in IIH patients is due to the loss of nerve fibers and retinal ganglion cells resulting from oxidative stress-associated prolonged swelling. The subarachnoid space connected to the optic nerve sheath undergoes structural changes due to alterations in the translaminar pressure gradient (difference between intraocular pressure and CSF pressure). Elevated ICP compresses the retrolaminar optic nerve and peripapillary scleral flange, causing deformation of the ppRPE/BM and adjacent sclera toward the vitreous [120–122]. Studies in this review have shown that in IIH patients, the configuration of the ppRPE/BM follows a U-shaped pattern around the optic nerve head, transitioning to a V-shape after interventions to lower CSF levels [19].

The earliest finding of raised ICP is optic disc swelling which takes about a week or 10 days to appear. However various diagnostic methods such as MRI, serum hormonal assay, axial length evaluation, pattern electroretinogram (PERG), and visually evoked potential (VEP) tests, can aid in detecting subclinical IIH. Liu et al. demonstrated that patients experiencing pulsatile tinnitus displayed several ocular and intracranial signs of IIH on MRI scans, such as optic nerve sheath enlargement, optic nerve tortuosity, posterior globe flattening, empty Sella, downward displacement of cerebellar tonsils into the foramen magnum, and slit-like lateral ventricles [123].According to a study by Prabhat and colleagues, hormonal abnormalities such as raised prolactin, decreased TSH, and decreased cortisol were found in 37.5% of patients with IIH [124]. Moreover, studies have shown that the mean PERG and VEP amplitudes were reduced in IIH patients compared to healthy individuals [125]. Madill et al.in their study reported a significant difference in globe shape and axial length between patients with IIH and control subjects [126].

Swelling of the optic disc and increased thickness of the RNFL are not exclusive indicators of IIH or increased ICP because they can also occur in other optic neuropathies like optic neuritis and AION. Nevertheless, various parameters that describe the shape of the optic nerve head, such as, optic nerve head volume, optic nerve cup volume, central optic nerve head thickness, volume of Bruch’s membrane opening region, bending energy, minimal rim width of Bruch’s membrane opening (BMO-MRW), surface area of BMO-MRW, and area of Bruch’s membrane opening, may aid in distinguishing between different optic neuropathies. In a study by Yadav et al., a 3D model of the optic nerve head was constructed using high-resolution OCT volume scans, and it was demonstrated that all of the aforementioned parameters, except for bending energy, exhibited differences between IIH, healthy controls, and optic neuritis [127]. Similarly, Kaufhold and colleagues employed volume scans to gauge optic nerve head volume in their study, revealing that 3D parameters such as optic nerve head volume and height could distinguish between IIH patients and controls [35]. These parameters were shown to be elevated in IIH patients even when the RNFL showed normal thickness, suggesting that it could serve as a potential marker of treatment efficacy and disease advancement [35]. Future studies employing OCT as a diagnostic tool for IIH could utilize the 3D optic nerve head parameters to differentiate IIH for other optic neuropathies such as glaucoma, optic neuritis, and AION.

The central retinal artery and ophthalmic artery traverse through the subarachnoid space and are influenced by changes in ICP [128]. Out of ten studies utilizing OCT-A as an imaging modality in IIH, five revealed a decrease in peripapillary vessel density [107, 108, 111–113], one demonstrated an increase in vessel density [109], and one found no disparity in vessel density between IIH patients and controls [110]. The decrease in vessel density seen in OCT-A can be due to mechanical compression of the capillary network caused by elevated ICP [129] or due to artifacts arising from the shadowing effect of fluid in papilledema artificially leading to decreased capillary density [129, 130]. Reduction in capillary vessel density has also been reported in other acute and chronic optic neuropathies such as optic neuritis, Leber’s hereditary optic neuropathy (LHON), optic atrophy and non-arteritic anterior ischemic optic neuropathy (NAION) [39]. In cases of optic neuritis and dominant optic atrophy, the decrease in vessel density may result from reduced metabolic demands caused by neuronal degeneration and the atrophy of the peripapillary RNFL and GC-IPL, which subsequently reduces blood flow through autoregulatory mechanisms [131]. However, LHON is a peripapillary microangiopathy that affects the endothelial and smooth muscle components of the blood vessel walls causing a significant reduction in the peripapillary capillary density [132]. In NAION, ischemic alterations due to dysfunctional vascular autoregulation may lead to the destruction of the capillaries [39].

**Fig. 7:**
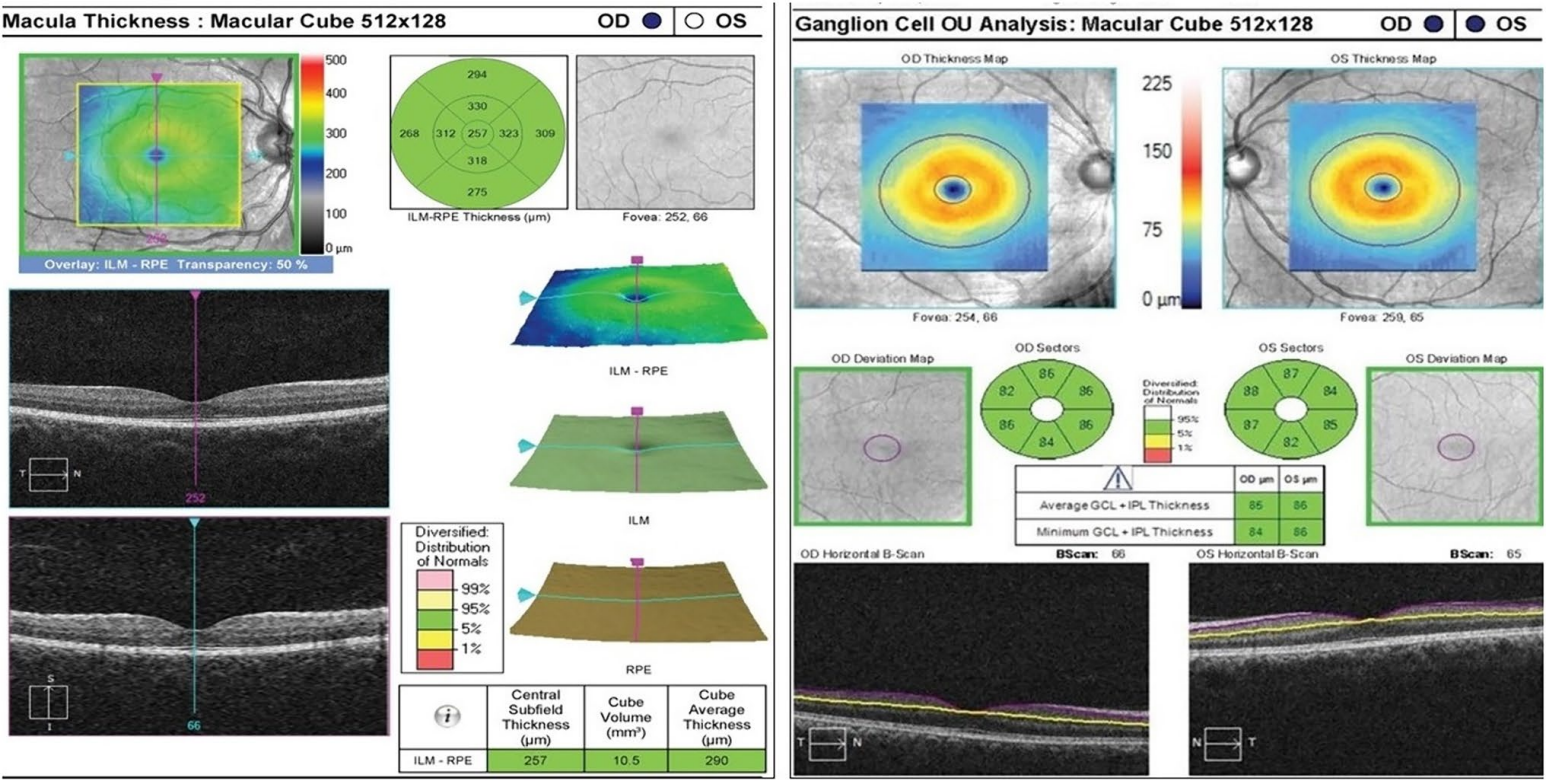
Optical coherence tomography (OCT) imaging test results showing A, macular thickness and B, ganglion cell complex (GCC) thickness

The OCT parameters such as RNFL thickness, macular GCC thickness, rim area, disc area, and cup volume are easily obtained through device software (Figs. 6 and 7). However, certain parameters like optic nerve head volume, optic nerve head height, optic nerve head shape, peripapillary Bruch’s membrane angle, anterior laminar surface depth, posterior laminar surface depth, and Bruch’s membrane opening require manual calculation using custom segmentation algorithms. Most OCT devices do not automatically provide these measures, limiting their practicality in routine clinical use. In addition, accurately segmenting the outer retinal boundary in the presence of papilledema can be challenging and may lead to inaccuracies [133]. Another crucial OCT parameter for distinguishing papilledema in IIH from optic disc edema caused by other factors is the ppRPE/BM shape changes. These changes have shown a correlation with ICP [96]. However, the practical application of using ppRPE/BM changes in guiding clinical therapy is hindered by the lack of a commercial method and the need for extensive image processing to identify the RPE/BM boundary beneath an enlarged optic nerve head, limiting the integration of ppRPE/BM changes into clinical decision-making. OCT-A is relatively newer imaging methods offering both structural and blood flow information within the retina and the choroid [134]. Given that recent studies using OCT-A in IIH have produced inconsistent findings regarding peripapillary vessel density, future research should concentrate on changes in the perifoveal capillary network. This is because IIH is linked to reduced blood flow in the ophthalmic and central retinal arteries [135].

This is the first systematic review to explore studies utilizing ocular imaging as a biomarker for IIH. One of the strengths is that it included 84 studies that used either OCT or OCT-A as the imaging modality in IIH patients. In addition, this systematic review employed study quality assessment tools to appraise the quality of the included studies. There were some limitations in the study. Most of the studies included in this review were case–control studies, with only two randomized control trials. This is because most studies focused on comparing the retinal and optic nerve head changes between IIH patients and healthy controls, thus adopting a case–control design. Another limitation of the study was that it only reviewed various OCT/OCT-A parameters in IIH and could not perform a meta-analysis of these common parameters. This initial step was necessary to understand the current studies and their reporting methods, which will facilitate future meta-analyses. Moreover, conducting a meta-analysis was not possible at this stage given the following reasons: (1) the studies had significantly different designs, methods, and levels of rigor, it might be inappropriate or misleading to statistically combine their results, (2) the outcomes were reported in diverse ways, making it difficult to aggregate the results meaningfully, and (3) some studies lacked sufficient data points for a robust meta-analysis, and some were of low quality or had a high risk of bias, which could lead to misleading conclusions if combined. A narrative approach allows for a more nuanced discussion of the quality and implications of each study. Finally, the specific research question or objective of the review was thought to be better addressed through a narrative synthesis, particularly because the question is broad or exploratory in nature. Another limitation was that our review comprised just ten studies examining the utility of OCT-A in individuals with IIH. This limited number of studies may be attributed to the fact that OCT-A is a relatively recent technology, and its effectiveness in a systemic condition like IIH has yet been firmly established.

To conclude, several OCT parameters have been demonstrated to be different in IIH patients compared to controls and retinal imaging may be useful as an efficient, non-invasive, and affordable biomarker for IIH patients.

### Supplementary Information

Below is the link to the electronic supplementary material.

Supplementary file1 (DOCX 26 KB)

Supplementary file2 (DOCX 109 KB)

## Data Availability

The data supporting the findings of this systematic review can be obtained from the corresponding author, Mallika Prem Senthil on request.
