# Supplementary material for: Exploring the utility of retinal optical coherence tomography as a biomarker for idiopathic intracranial hypertension: a systematic review
Source: J Neurol. 2024 Jun 10;271(8):4769–93. doi: 10.1007/s00415-024-12481-3 (PMC11319609; doi:10.1007/s00415-024-12481-3)
Supplement: Supplementary file 1 — Supplementary file1 (DOCX 26 KB) [file 415_2024_12481_MOESM1_ESM.docx]

Journal of Neurology

Title: **Exploring the utility of retinal optical coherence tomography as a biomarker for idiopathic intracranial hypertension: a systematic review**

Mallika Prem Senthil PhD^1^, Ranjay Chakraborty PhD^1^, Jose Estevez Bordon B.MedSci (VisSci) M.Optom^1^, Paul A. Constable PhD^1^, Shannon Brown BA (Psych), GradDip (InfoMgmt) ^1^, Saumya Anand B.MedSci (VisSci) M.Optom^1^, Dalia Al-Dasooqi B.MedSci (VisSci) M.Optom^1,^ Simu Simon FRANZCO^2^

^1^College of Nursing and Health Sciences, Caring Futures Institute, Flinders University, Bedford Park, Adelaide, South Australia, Australia

^2^Central Library, Flinders University, Bedford Park, Adelaide, South Australia, Australia

^3^University of Adelaide, Adelaide, South Australia, Australia

**Corresponding author**

Email: [mallika.premsenthil@flinders.edu.au](mailto:mallika.premsenthil@flinders.edu.au)

**Supplementary material 1. Search terms that were used for the different databases in this systematic review.**

**Database: Medline**

| Ovid MEDLINE(R) and Epub Ahead of Print, In-Process, In-Data-Review & Other Non-Indexed Citations, Daily and Versions <1946 to January 22, 2024> | | |
| --- | --- | --- |
| 1 | optical imaging/ or narrow band imaging/ or tomography, optical/ or tomography, optical coherence/ or transillumination/ or Tomography, Optical Coherence/ | 67871 |
| 2 | diagnostic imaging/ or exp image interpretation, computer-assisted/ or exp imaging, three-dimensional/ or tomography/ or Computed Tomography Angiography/ | 716138 |
| 3 | (transillumination or tomograph*).tw,kf. | 547310 |
| 4 | (imag* adj3 (computer* or three-dimension* or 3D or 3-d or interpret* or diagnos* or fluorescence)).tw,kf. | 155718 |
| 5 | or/1-4 | 1118844 |
| 6 | exp Retina/ | 154731 |
| 7 | (retina* or fundus or choroid or macular or fovea or amacrine or photorecptor*).tw,kf. | 295234 |
| 8 | or/6-7 | 331724 |
| 9 | 5 and 8 | 52232 |
| 10 | intracranial hypertension/ or posterior leukoencephalopathy syndrome/ or pseudotumor cerebri/ | 11675 |
| 11 | (intra?cranial or intra cranial or inter?cranial or inter cranial or brain* or cerebral*).tw,kf. | 1618315 |
| 12 | (hypertens* or pressur*).tw,kf. | 1383472 |
| 13 | (benign* or idiopathic* or secondary).tw,kf. | 1286309 |
| 14 | 11 and 12 and 13 | 12027 |
| 15 | ((pseudotumor or pseudo tumor* or pseudotumour or pseudo tumour*) adj3 (cerebr* or brain*)).tw,kf. | 2007 |
| 16 | ((otitic or otitis) adj5 hydroceph*).tw,kf. | 91 |
| 17 | (meningeal adj3 hydrop*).tw,kf. | 6 |
| 18 | (Noninfect* adj3 meningitis).tw,kf. | 22 |
| 19 | (posterior adj2 leukoencephalopath*).tw,kf. | 710 |
| 20 | Nonne* syndrome.tw,kf. | 3 |
| 21 | (BIH or IIH).tw,kf. | 3331 |
| 22 | 10 or 14 or 15 or 16 or 17 or 18 or 19 or 20 or 21 | 23030 |
| 23 | 9 and 22 | 295 |
| 24 | exp animals/ not humans.sh. | 5189493 |
| 25 | 23 not 24 | 291 |

**Database: Embase**

| Embase <1974 to 2024 January 22> | | |
| --- | --- | --- |
| 1 | fluorescence imaging/ or narrow band imaging/ or optical tomography/ or optical coherence tomography/ or transillumination/ | 124004 |
| 2 | computer assisted tomography/ or four dimensional computed tomography/ or high resolution computer tomography/ or computed tomographic angiography/ or diagnostic imaging/ | 1229583 |
| 3 | (transillumination or tomograph*).tw,kw. | 677992 |
| 4 | (imag* adj3 (computer* or three-dimension* or 3D or 3-d or interpret* or diagnos* or fluorescence)).tw,kw. | 204411 |
| 5 | or/1-4 | 1708484 |
| 6 | exp retina/ | 157225 |
| 7 | (retina* or fundus or choroid or macular or fovea or amacrine or photorecptor*).tw,kw. | 365396 |
| 8 | or/6-7 | 387634 |
| 9 | 5 and 8 | 70610 |
| 10 | intracranial hypertension/ or idiopathic intracranial hypertension/ | 23568 |
| 11 | brain pseudotumor/ | 4502 |
| 12 | (intra?cranial or intra cranial or inter?cranial or inter cranial or brain* or cerebral*).tw,kf. | 2106887 |
| 13 | (hypertens* or pressur*).tw,kf. | 1898386 |
| 14 | (benign* or idiopathic* or secondary).tw,kf. | 1815221 |
| 15 | 12 and 13 and 14 | 20491 |
| 16 | ((pseudotumor or pseudo tumor* or pseudotumour or pseudo tumour*) adj3 (cerebr* or brain*)).tw,kf. | 2755 |
| 17 | ((otitic or otitis) adj5 hydroceph*).tw,kf. | 81 |
| 18 | (meningeal adj3 hydrop*).tw,kf. | 2 |
| 19 | (Noninfect* adj3 meningitis).tw,kf. | 27 |
| 20 | (posterior adj2 leukoencephalopath*).tw,kf. | 1056 |
| 21 | Nonne* syndrome.tw,kf. | 4 |
| 22 | (BIH or IIH).tw,kf. | 4892 |
| 23 | 10 or 11 or 15 or 16 or 17 or 18 or 19 or 20 or 21 or 22 | 44417 |
| 24 | 9 and 23 | 609 |
| 25 | (exp animal/ or exp invertebrate/ or nonhuman/ or animal experiment/ or animal tissue/ or animal model/ or exp plant/ or exp fungus/) not (exp human/ or human tissue/) | 7832301 |
| 26 | 24 not 25 | 598 |

**Database: Emcare**

| Ovid Emcare <1995 to 2024 Week 03> | | |
| --- | --- | --- |
| 1 | diagnostic imaging/ or transillumination/ or narrow band imaging/ or exp computer assisted tomography/ or exp computed tomographic angiography/ | 281950 |
| 2 | (imag* adj3 (computer* or three-dimension* or 3D or 3-d or interpret* or diagnos* or fluorescence)).tw,kw. | 51646 |
| 3 | or/1-2 | 318194 |
| 4 | exp retina/ | 10516 |
| 5 | (retina* or fundus or choroid or macular or fovea or amacrine or photorecptor*).tw,kw. | 41509 |
| 6 | or/4-5 | 43004 |
| 7 | 3 and 6 | 1739 |
| 8 | intracranial hypertension/ or idiopathic intracranial hypertension/ | 4020 |
| 9 | (intra?cranial or intra cranial or inter?cranial or inter cranial or brain* or cerebral*).tw,kw. | 360562 |
| 10 | (hypertens* or pressur*).tw,kw. | 372077 |
| 11 | (benign* or idiopathic* or secondary).tw,kw. | 373362 |
| 12 | 9 and 10 and 11 | 3454 |
| 13 | ((pseudotumor or pseudo tumor* or pseudotumour or pseudo tumour*) adj3 (cerebr* or brain).tw,kw. | 298 |
| 14 | ((otitic or otitis) adj5 hydroceph*).tw,kw. | 18 |
| 15 | (meningeal adj3 hydrop*).tw,kw. | 0 |
| 16 | (Noninfect* adj3 meningitis).tw,kw. | 9 |
| 17 | (posterior adj2 leukoencephalopath*).tw,kw. | 156 |
| 18 | Nonne* syndrome.tw,kw. | 0 |
| 19 | (BIH or IIH).tw,kw. | 1032 |
| 20 | 8 or 12 or 13 or 14 or 15 or 16 or 17 or 18 or 19 | 7748 |
| 21 | 7 and 20 | 19 |
| 22 | exp animal/ not human/ | 289865 |
| 23 | 21 not 22 | 19 |

**Database: Scopus**

( TITLE-ABS-KEY ( ( ( ( intra?cranial OR intra AND cranial OR inter?cranial OR inter AND cranial OR brain* OR cerebral* ) AND ( hypertens* OR pressur* ) AND ( benign* OR idiopathic* OR secondary ) ) OR ( ( otitic OR otitis ) W/5 hydroceph* ) OR ( meningeal W/3 hydrop* ) OR ( noninfect* W/3 meningitis ) OR ( posterior W/2 leukoencephalopath* ) OR ( nonne* AND syndrome OR bih OR iih ) ) ) AND TITLE-ABS-KEY ( ( transillumination OR tomograph* OR ( imag* W/3 ( computer* OR three-dimension* OR 3d OR "3-d" OR interpret* OR diagnos* OR fluorescence ) ) ) ) ) AND ( EXCLUDE ( SUBJAREA , "VETE" ) OR EXCLUDE ( SUBJAREA , "AGRI" ) OR EXCLUDE ( SUBJAREA , "PHYS" ) ) AND ( LIMIT-TO ( DOCTYPE , "re" ) OR LIMIT-TO ( DOCTYPE , "ar" ) ) AND ( LIMIT-TO ( LANGUAGE , "English" ) )

**Database: ProQuest Central**

noft(((retina* OR fundus OR choroid OR macular OR fovea OR amacrine OR photorecptor*) AND (transillumination OR tomograph* OR (imag* NEAR/3 (computer* OR three-dimension* OR 3D OR "3-d" OR interpret* OR diagnos* OR fluorescence))))) AND noft((((intra?cranial OR intra cranial OR inter?cranial OR inter cranial OR brain* OR cerebral*) AND (hypertens* OR pressur*) AND (benign* OR idiopathic* OR secondary)) OR ((otitic OR otitis) NEAR/5 hydroceph*) OR (meningeal NEAR/3 hydrop*) OR (Noninfect* NEAR/3 meningitis) OR (posterior NEAR/2 leukoencephalopath*) OR (Nonne* syndrome OR BIH OR IIH)))

**Database: Cochrane Central**

(retina* OR fundus OR choroid OR macular OR fovea OR amacrine OR photorecptor*)

(((intra?cranial OR intra cranial OR inter?cranial OR inter cranial OR brain* OR cerebral*) AND (hypertens* OR pressur*) AND (benign* OR idiopathic* OR secondary)) OR ((otitic OR otitis) NEAR/5 hydroceph*) OR (meningeal NEAR/3 hydrop*) OR (Noninfect* NEAR/3 meningitis) OR (posterior NEAR/2 leukoencephalopath*) OR (“Nonne's syndrome” OR BIH OR IIH))

**Final search string**

(((image or imaging) NEAR/3 (computer OR three-dimensional OR 3D OR interpret* OR diagnostic OR fluorescence))):ti,ab,kw AND ((retina* OR fundus OR choroid OR macular OR fovea OR amacrine OR photorecptor*)):ti,ab,kw AND ((((intra?cranial OR intra cranial OR inter?cranial OR inter cranial OR brain* OR cerebral*) AND (hypertens* OR pressur*) AND (benign* OR idiopathic* OR secondary)) OR ((otitic OR otitis) NEAR/5 hydroceph*) OR (meningeal NEAR/3 hydrop*) OR (Noninfect* NEAR/3 meningitis) OR (posterior NEAR/2 leukoencephalopath*) OR (“Nonne's syndrome” OR BIH OR IIH))):ti,ab,kw
