# Supplementary material for: Exploring the utility of retinal optical coherence tomography as a biomarker for idiopathic intracranial hypertension: a systematic review
Source: J Neurol. 2024 Jun 10;271(8):4769–93. doi: 10.1007/s00415-024-12481-3 (PMC11319609; doi:10.1007/s00415-024-12481-3)
Supplement: Supplementary file 2 — Supplementary file2 (DOCX 109 KB) [file 415_2024_12481_MOESM2_ESM.docx]

Journal of Neurology

Title: **Exploring the utility of retinal optical coherence tomography as a biomarker for idiopathic intracranial hypertension: a systematic review**

Mallika Prem Senthil PhD^1^, Ranjay Chakraborty PhD^1^, Jose Estevez Bordon B.MedSci (VisSci) M.Optom^1^, Paul A. Constable PhD^1^, Shannon Brown BA (Psych), GradDip (InfoMgmt) ^1^, Saumya Anand B.MedSci (VisSci) M.Optom^1^, Dalia Al-Dasooqi B.MedSci (VisSci) M.Optom^1,^ Simu Simon FRANZCO^2^

^1^College of Nursing and Health Sciences, Caring Futures Institute, Flinders University, Bedford Park, Adelaide, South Australia, Australia

^2^Central Library, Flinders University, Bedford Park, Adelaide, South Australia, Australia

^3^University of Adelaide, Adelaide, South Australia, Australia

**Corresponding author**

Email: [mallika.premsenthil@flinders.edu.au](mailto:mallika.premsenthil@flinders.edu.au)

**Supplemental material 2. Quality Assessment of Case-control studies using the National Institute of Health Study Quality Assessment Tool**

| **Authors** | **Year** | **1** | **2** | **3** | **4** | **5** | **6** | **7** | **8** | **9** | **10** | **11** | **12** |
| --- | --- | --- | --- | --- | --- | --- | --- | --- | --- | --- | --- | --- | --- |
| Rebolleda et al | 2009 | 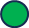 | 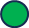 | 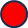 | 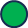 | 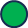 | 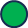 | 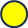 | 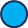 | 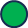 | 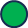 | 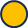 | 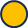 |
| Jensen et al | 2010 | 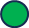 | 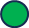 | 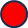 | 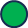 | 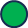 | 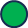 | 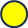 | 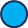 | 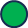 | 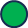 | 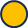 | 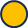 |
| Sibony et al | 2011 | 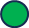 | 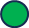 | 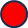 | 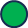 | 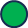 | 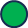 | 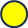 | 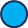 | 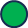 | 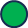 | 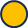 | 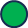 |
| Skau et al | 2011 | 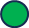 | 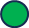 | 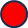 | 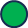 | 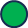 | 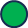 | 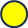 | 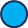 | 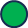 | 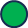 | 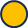 | 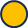 |
| Skau et al | 2011 | 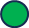 | 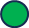 | 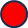 | 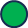 | 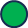 | 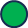 | 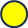 | 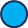 | 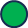 | 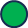 | 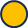 | 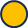 |
| Kaufhold et al | 2012 | 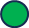 | 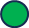 | 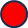 | 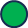 | 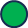 | 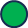 | 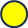 | 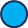 | 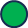 | 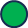 | 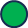 | 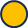 |
| Yri et al | 2012 | 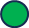 | 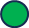 | 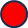 | 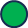 | 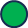 | 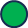 | 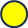 | 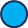 | 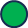 | 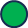 | 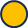 | 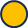 |
| Skau et al | 2013 | 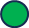 | 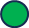 | 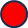 | 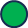 | 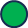 | 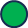 | 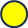 | 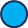 | 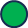 | 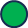 | 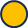 | 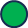 |
| Fard et al | 2014 | 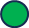 | 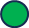 | 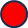 | 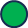 |  |  |  |  |  |  |  |  |
| Monteiro et al | 2014 |  |  |  |  |  |  |  |  |  |  |  |  |
| Afonso et al | 2015 |  |  |  |  |  |  |  |  |  |  |  |  |
| Chang et al | 2015 |  |  |  |  |  |  |  |  |  |  |  |  |
| Moss et al | 2015 |  |  |  |  |  |  |  |  |  |  |  |  |
| Labib et al | 2015 |  |  |  |  |  |  |  |  |  |  |  |  |
| Goldhagen et al | 2015 |  |  |  |  |  |  |  |  |  |  |  |  |
| Albrecht et al | 2017 |  |  |  |  |  |  |  |  |  |  |  |  |
| Aojula et al | 2018 |  |  |  |  |  |  |  |  |  |  |  |  |
| Park et al | 2018 |  |  |  |  |  |  |  |  |  |  |  |  |
| Eren et al | 2019 |  |  |  |  |  |  |  |  |  |  |  |  |
| Fard et al | 2019 |  |  |  |  |  |  |  |  |  |  |  |  |
| Huang-Link et al | 2019 |  |  |  |  |  |  |  |  |  |  |  |  |
| Pasaoglu et al | 2019 |  |  |  |  |  |  |  |  |  |  |  |  |
| Tüntaş Bilen et al | 2019 |  |  |  |  |  |  |  |  |  |  |  |  |
| Wall et al | 2019 |  |  |  |  |  |  |  |  |  |  |  |  |
| Merticariu et al | 2019 |  |  |  |  |  |  |  |  |  |  |  |  |
| Dreesbach et al | 2020 |  |  |  |  |  |  |  |  |  |  |  |  |
| Tatar et al | 2020 |  |  |  |  |  |  |  |  |  |  |  |  |
| Ozdemir et al | 2020 |  |  |  |  |  |  |  |  |  |  |  |  |
| Kabatas et al | 2021 |  |  |  |  |  |  |  |  |  |  |  |  |
| Nogueira et al | 2021 |  |  |  |  |  |  |  |  |  |  |  |  |
| Panyala et al | 2021 |  |  |  |  |  |  |  |  |  |  |  |  |
| Kaya et al | 2021 |  |  |  |  |  |  |  |  |  |  |  |  |
| Jacobsen et al | 2022 |  |  |  |  |  |  |  |  |  |  |  |  |
| Banerjee et al | 2022 |  |  |  |  |  |  |  |  |  |  |  |  |
| Chonsui et al | 2022 |  |  |  |  |  |  |  |  |  |  |  |  |
| Kaya et al | 2023 |  |  |  |  |  |  |  |  |  |  |  |  |
| Yalcinkaya Cakir et al | 2023 |  |  |  |  |  |  |  |  |  |  |  |  |
| Wang et al | 2023 |  |  |  |  |  |  |  |  |  |  |  |  |
| Pahuja et al | 2023 |  |  |  |  |  |  |  |  |  |  |  |  |
| Kwapong et al | 2023 |  |  |  |  |  |  |  |  |  |  |  |  |
| Srija et al | 2024 |  |  |  |  |  |  |  |  |  |  |  |  |
| Wang et al | 2024 |  |  |  |  |  |  |  |  |  |  |  |  |

= Yes; = No; = Not applicable; = Cannot determine; = Not reported

**1** = Was the research question clearly stated?

**2** = Was the study population clearly specified and defined?

**3** = Did the authors include a sample size justification?

**4** = Were controls selected or recruited from the same or similar population that gave rise to the cases (including the same timeframe)?

**5** = Were the definitions, inclusion and exclusion criteria used to identify or select cases and controls valid, reliable, and implemented consistently across all study participants?

**6** = Were the cases clearly defined and differentiated from controls?

**7** = If less than 100 percent of eligible cases and/or controls were selected for the study, were the cases and/or controls randomly selected from those eligible?

**8** = Was there use of concurrent controls?

**9** = Were the investigators able to confirm that the exposure/risk occurred prior to the development of the condition or event that defined a participant as a case?

**10** = Were the measures of exposure/risk clearly defined, valid, reliable, and implemented consistently across all study participants?

**11** = Were the assessors of exposure/risk blinded to the case or control status of participants?

**12** = Were key potential confounding variables measured and adjusted statistically in the analyses?

**Quality Assessment of Observational Cohort and Cross-Sectional Studies using the National Institute of Health Study Quality Assessment Tools**

| **Author** | **Year** | **1** | **2** | **3** | **4** | **5** | **6** | **7** | **8** | **9** | **10** | **11** | **12** | **13** | **14** |
| --- | --- | --- | --- | --- | --- | --- | --- | --- | --- | --- | --- | --- | --- | --- | --- |
| Scott et al | 2010 |  |  |  |  |  |  |  |  |  |  |  |  |  |  |
| Sinclair et al | 2010 |  |  |  |  |  |  |  |  |  |  |  |  |  |  |
| Waisbourd et al | 2011 |  |  |  |  |  |  |  |  |  |  |  |  |  |  |
| Marzoli et al | 2013 |  |  |  |  |  |  |  |  |  |  |  |  |  |  |
| Auinger et al | 2014 |  |  |  |  |  |  |  |  |  |  |  |  |  |  |
| Chen et al | 2015 |  |  |  |  |  |  |  |  |  |  |  |  |  |  |
| Sibony et al | 2015 |  |  |  |  |  |  |  |  |  |  |  |  |  |  |
| Starks et al | 2016 |  |  |  |  |  |  |  |  |  |  |  |  |  |  |
| Kupersmith et al | 2017 |  |  |  |  |  |  |  |  |  |  |  |  |  |  |
| Saenz et al | 2017 |  |  |  |  |  |  |  |  |  |  |  |  |  |  |
| Wang et al | 2017 |  |  |  |  |  |  |  |  |  |  |  |  |  |  |
| Saenz et al | 2017 |  |  |  |  |  |  |  |  |  |  |  |  |  |  |
| Shiels et al | 2018 |  |  |  |  |  |  |  |  |  |  |  |  |  |  |
| Onder et al | 2019 |  |  |  |  |  |  |  |  |  |  |  |  |  |  |
| Bahnasy et al | 2020 |  |  |  |  |  |  |  |  |  |  |  |  |  |  |
| Vijay et al | 2020 |  |  |  |  |  |  |  |  |  |  |  |  |  |  |
| Chen et al | 2020 |  |  |  |  |  |  |  |  |  |  |  |  |  |  |
| Flowers et al | 2021 |  |  |  |  |  |  |  |  |  |  |  |  |  |  |
| Bingol Kiziltuncet et al | 2021 |  |  |  |  |  |  |  |  |  |  |  |  |  |  |
| Carey et al | 2021 |  |  |  |  |  |  |  |  |  |  |  |  |  |  |
| Kohil et al | 2021 |  |  |  |  |  |  |  |  |  |  |  |  |  |  |
| Reggie et al | 2021 |  |  |  |  |  |  |  |  |  |  |  |  |  |  |
| Rodriguez et al | 2021 |  |  |  |  |  |  |  |  |  |  |  |  |  |  |
| Touze et al | 2021 |  |  |  |  |  |  |  |  |  |  |  |  |  |  |
| Wibroe et al | 2021 |  |  |  |  |  |  |  |  |  |  |  |  |  |  |
| Inam et al | 2022 |  |  |  |  |  |  |  |  |  |  |  |  |  |  |
| Thaller et al | 2022 |  |  |  |  |  |  |  |  |  |  |  |  |  |  |
| Rehman et al | 2022 |  |  |  |  |  |  |  |  |  |  |  |  |  |  |
| Rehman et al | 2022 |  |  |  |  |  |  |  |  |  |  |  |  |  |  |
| Sood et al | 2022 |  |  |  |  |  |  |  |  |  |  |  |  |  |  |
| Vosoughi et al | 2022 |  |  |  |  |  |  |  |  |  |  |  |  |  |  |
| Attia et al | 2023 |  |  |  |  |  |  |  |  |  |  |  |  |  |  |
| El-Haddad et al | 2023 |  |  |  |  |  |  |  |  |  |  |  |  |  |  |
| Kaya Tutar et al | 2023 |  |  |  |  |  |  |  |  |  |  |  |  |  |  |
| Thaller et al | 2023 |  |  |  |  |  |  |  |  |  |  |  |  |  |  |
| Thaller et al | 2023 |  |  |  |  |  |  |  |  |  |  |  |  |  |  |
| Xie et al | 2023 |  |  |  |  |  |  |  |  |  |  |  |  |  |  |
| Bassi et al | 2024 |  |  |  |  |  |  |  |  |  |  |  |  |  |  |

= **Yes**; = **No**; = **Not applicable**; = **Cannot determine**; = **Not reported**

**1** = Was the research question or objective in this paper clearly stated?

**2** = Was the study population clearly specified and defined?

**3** = Was the participation rate of eligible persons at least 50%?

**4** = Were all the subjects selected or recruited from the same or similar populations?

**5** = Was a sample size justification, power description, or variance and effect estimates provided?

**6** = For the analyses in this paper, were the exposure(s) of interest measured prior to the outcome(s) being measured?

**7** = Was the timeframe sufficient so that one could reasonably expect to see an association between exposure and outcome if it existed?

**8** = For exposures that can vary in amount or level, did the study examine different levels of the exposure as related to the outcome?

**9** = Were the exposure measures (independent variables) clearly defined, valid, reliable, and implemented consistently across all study participants?

**10** = Was the exposure(s) assessed more than once over time?

**11**= Were the outcome measures (dependent variables) clearly defined, valid, reliable, and implemented consistently across all study participants?

**12** = Were the outcome assessors blinded to the exposure status of participants?

**13** = Was loss to follow-up after baseline 20% or less?

**14** = Were key potential confounding variables measured and adjusted statistically for their impact on the relationship between exposure(s) and outcome(s)?

**Quality Assessment of Controlled Intervention Studies using the National Institute of Health Study Quality Assessment Tool**

| **Author** | **Year** | **1** | **2** | **3** | **4** | **5** | **6** | **7** | **8** | **9** | **10** | **11** | **12** | **13** | **14** |
| --- | --- | --- | --- | --- | --- | --- | --- | --- | --- | --- | --- | --- | --- | --- | --- |
| OCT sub study committee | 2015 |  |  |  |  |  |  |  |  |  |  |  |  |  |  |
| Banik et al | 2019 |  |  |  |  |  |  |  |  |  |  |  |  |  |  |

= **Yes**; = **No**; = **Not applicable**; = **Cannot determine**; = **Not reported**

**1** = Was the study described as randomized, a randomized trial, a randomized clinical trial, or an RCT?

**2** = Was the method of randomization adequate (i.e., use of randomly generated assignment)?

**3** =Was the treatment allocation concealed (so that assignments could not be predicted)?

**4** = Were study participants and providers blinded to treatment group assignment?

**5** = Were the people assessing the outcomes blinded to the participants' group assignments?

**6** =Were the groups similar at baseline on important characteristics that could affect outcomes (e.g., demographics, risk factors, co-morbid conditions)?

**7** =Was the overall drop-out rate from the study at endpoint 20% or lower of the number allocated to treatment?

**8** =Was the differential drop-out rate (between treatment groups) at endpoint 15 percentage points or lower?

**9** = Was there high adherence to the intervention protocols for each treatment group?

**10** = Were other interventions avoided or similar in the groups (e.g., similar background treatments)?

**11** =Were outcomes assessed using valid and reliable measures, implemented consistently across all study participants?

**12** = Did the authors report that the sample size was sufficiently large to be able to detect a difference in the main outcome between groups with at least 80% power?

**13** = Were outcomes reported or subgroups analyzed prespecified (i.e., identified before analyses were conducted)?

**14** =Were all randomized participants analyzed in the group to which they were originally assigned, i.e., did they use an intention-to-treat analysis?

**Quality Assessment of Before-After (Pre-Post) Studies with No Control Group using the National Institute of Health Study Quality Assessment Tool**

| **Author** | **Year** | **1** | **2** | **3** | **4** | **5** | **6** | **7** | **8** | **9** | **10** | **11** | **12** |
| --- | --- | --- | --- | --- | --- | --- | --- | --- | --- | --- | --- | --- | --- |
| Sibony et al | 2014 |  |  |  |  |  |  |  |  |  |  |  |  |
| Dinkin et al | 2017 |  |  |  |  |  |  |  |  |  |  |  |  |
| Gampa et al | 2017 |  |  |  |  |  |  |  |  |  |  |  |  |

= **Yes**; = **No**; = **Not applicable**; = **Cannot determine**; = **Not reported**

**1** = Was the study question or objective clearly stated?

**2** = Were eligibility/selection criteria for the study population prespecified and clearly described?

**3** = Were the participants in the study representative of those who would be eligible for the test/service/intervention in the general or clinical population of interest?

**4** = Were all eligible participants that met the prespecified entry criteria enrolled?

**5** = Was the sample size sufficiently large to provide confidence in the findings?

**6** = Was the test/service/intervention clearly described and delivered consistently across the study population?

**7** = Were the outcome measures prespecified, clearly defined, valid, reliable, and assessed consistently across all study participants?

**8** = Were the people assessing the outcomes blinded to the participants' exposures/interventions?

**9** = Was the loss to follow-up after baseline 20% or less? Were those lost to follow-up accounted for in the analysis?

**10** = Did the statistical methods examine changes in outcome measures from before to after the intervention? Were statistical tests done that provided p values for the pre-to-post changes?

**11** = Were outcome measures of interest taken multiple times before the intervention and multiple times after the intervention (i.e., did they use an interrupted time-series design)?

**12** = If the intervention was conducted at a group level (e.g., a whole hospital, a community, etc.) did the statistical analysis take into account the use of individual-level data to determine effects at the group level?
